# Genetic polymorphisms influencing deferasirox pharmacokinetics, efficacy, and adverse drug reactions: a systematic review and meta-analysis

**DOI:** 10.3389/fphar.2023.1069854

**Published:** 2023-05-16

**Authors:** Kittika Yampayon, Puree Anantachoti, Bunchai Chongmelaxme, Varalee Yodsurang

**Affiliations:** ^1^ Department of Pharmacology and Physiology, Faculty of Pharmaceutical Sciences, Chulalongkorn University, Bangkok, Thailand; ^2^ Social and Administrative Pharmacy Department, Faculty of Pharmaceutical Sciences, Chulalongkorn University, Bangkok, Thailand; ^3^ Preclinical Toxicity and Efficacy, Assessment of Medicines and Chemicals Research Unit, Chulalongkorn University, Bangkok, Thailand

**Keywords:** deferasirox (DFX), pharmacogenomics (PGx), pharmacokinetics, pharmacodynamics, systematic reviews, meta-analysis

## Abstract

**Objective:** Deferasirox is an iron-chelating agent prescribed to patients with iron overload. Due to the interindividual variability of deferasirox responses reported in various populations, this study aims to determine the genetic polymorphisms that influence drug responses.

**Methods:** A systematic search was performed from inception to March 2022 on electronic databases. All studies investigating genetic associations of deferasirox in humans were included, and the outcomes of interest included pharmacokinetics, efficacy, and adverse drug reactions. Fixed- and random-effects model meta-analyses using the ratio of means (ROM) were performed.

**Results:** Seven studies involving 367 participants were included in a meta-analysis. The results showed that subjects carrying the A allele (AG/AA) of *ABCC2* rs2273697 had a 1.23-fold increase in deferasirox C_max_ (ROM = 1.23; 95% confidence interval [CI]:1.06–1.43; *p* = 0.007) and a lower Vd (ROM = 0.48; 95% CI: 0.36–0.63; *p* < 0.00001), compared to those with GG. A significant attenuated area under the curve of deferasirox was observed in the subjects with *UGT1A3* rs3806596 AG/GG by 1.28-fold (ROM = 0.78; 95% CI: 0.60–0.99; *p* = 0.04). In addition, two SNPs of *CYP24A1* were also associated with the decreased C_trough_: rs2248359 CC (ROM = 0.50; 95% CI: 0.29–0.87; *p* = 0.01) and rs2585428 GG (ROM = 0.47; 95% CI: 0.35–0.63; *p* < 0.00001). Only rs2248359 CC was associated with decreased C_min_ (ROM = 0.26; 95% CI: 0.08–0.93; *p* = 0.04), while rs2585428 GG was associated with a shorter half-life (ROM = 0.44; 95% CI: 0.23–0.83; *p* = 0.01).

**Conclusion:** This research summarizes the current evidence supporting the influence of variations in genes involved with drug transporters, drug-metabolizing enzymes, and vitamin D metabolism on deferasirox responses.

## 1 Introduction

Deferasirox (DFX) is an iron chelator approved for the treatment of iron overload (Porter and Viprakasit, 2014), and widely used in patients with transfusion-dependent anemia, such as thalassemia, sickle cell disease (SCD), and myelodysplastic syndromes (MDS). This excess iron is stored in the form of free iron and deposits into various organs, commonly the liver, heart, and endocrine glands, leading to organ damage and consequence complications (Porter and Viprakasit, 2014). Serum ferritin (SF) level generally correlates with the amount of stored iron in the body and is frequently used to monitor the body’s iron (World Health Organization, 2020; World Health Organization, 2021). Other indicators reflecting the iron level accumulated in the organs are liver iron concentration (LIC) (Telfer et al., 2000), cardiac T2* and R2* (1/T2*) (Ghugre et al., 2006; Bayraktaroğlu et al., 2011), which are measured using magnetic resonance imaging. The reduced T2* value is correlated with higher iron content, deterioration of left ventricular function, and risk of death (Borgna-Pignatti et al., 2004; Bayraktaroğlu et al., 2011; Fucharoen et al., 2014). Severe iron overload is defined as LIC >15 mg Fe/g liver dry weight, cardiac T2* MRI <10 ms, or SF > 2,500 ng/mL (Modell et al., 2000; Porter and Viprakasit, 2014; Shenoy et al., 2014). However, chelation therapy is started when SF exceeded 1,000 ng/mL or LIC >7 mg Fe/g liver dry weight to prevent the consequences of iron overload and prolonged survival (Fucharoen et al., 2014; Porter and Viprakasit, 2014).

As the pharmacological activity of DFX lowers excess iron, DFX binds to the iron. Then, the DFX-iron complex is predominantly eliminated by the hepatobiliary system and excreted via the fecal route (Waldmeier et al., 2010; Chalmers and Shammo, 2016). DFX induces negative iron balance, a decrease in SF, and reduced iron accumulation in the liver and heart (Cappellini et al., 2006; Pennell et al., 2011). In addition, DFX decreases the risk of organ damage due to reactive oxygen species (ROS) resulting from an increase in labile plasma iron and non-transferrin-bound iron (Galanello et al., 2012). Glucuronidation is the primary metabolic pathway for DFX, mainly by UDP glucuronosyltransferase family 1 member A1 (UGT1A1) and, to a lesser extent, by UDP glucuronosyltransferase family 1 member A3 (UGT1A3) (Novartis Pharmaceuticals Canada Inc., 2022). DFX and its metabolites are transported from the liver hepatocyte to the biliary system via multidrug resistance-associated protein 2 (MRP2), then excreted into the feces (Waldmeier et al., 2010). The cytochrome P450 enzymes (CYPs) play minor roles in the oxidative metabolism of DFX by CYP1A and CYP2D6.

Previous studies have reported variability in response to DFX ranging from 25.7% to 67.7% (Cappellini et al., 2006; Porter et al., 2008; Taher et al., 2011; Viprakasit et al., 2013; Cusato et al., 2015; Allegra et al., 2019). The number of pharmacogenetic studies explaining the genetic effects on interindividual differences in DFX responses has been increasing. Several single nucleotide polymorphisms (SNPs) involved with pharmacokinetics and pharmacodynamics of DFX and thalassemia disease have been investigated. To date, none of these studies have provided comprehensive information on the associations between DFX and their genetic responses. It is still unclear which SNPs influence DFX outcomes. This study aims to determine the associations between genetic polymorphisms and DFX outcomes, including pharmacokinetic (PK), iron-chelating efficacy, and adverse drug reactions (ADRs).

## 2 Methods

### 2.1 Data sources and search strategy

A systematic search was performed from inception to March 2022 in eight databases: PubMed, Embase, Cochrane CENTRAL, ClinicalTrials.gov, PharmGKB, GWAS Catalog, OpenGrey, and Thai Thesis Database. All the keywords used are presented in Supplementary Table S1. The references for retrieved articles were also examined to explore additional studies that were not indexed in the aforementioned databases.

### 2.2 Study selection

This review was conducted in accordance with the Preferred Reporting Items for Systematic Reviews and Meta-Analyses (PRISMA) guidelines (Moher et al., 2009) (Supplementary Table S2). All the randomized controlled trials (RCTs), cohort, and case-control studies that investigated the impact of genetic polymorphisms of DFX in humans were identified, and the outcomes of interest were 1) PK parameters, including AUC, C_max_, C_min_, C_trough_, t_1/2_, T_max_, and Vd; 2) Iron chelation efficacy that represented the amount of iron accumulated in the body; SF, LIC, liver stiffness (LS), hepatic T2*, and cardiac T2*; and 3) ADRs relevant to the results of liver and renal function tests. Initially, the titles and abstracts were screened to identify potential studies. Subsequently, the full texts were assessed by two investigators (KY and VY), and all disagreements between the investigators were resolved by a third reviewer (BC). The review protocol was registered in the PROSPERO database (no. CRD42021253045).

### 2.3 Data extraction and quality assessment

Data extraction was undertaken by KY and VY using a standardized form. The extracted data included the author’s name, year of publication, country of study setting, study design, patient characteristics (e.g., ethnicity, types of subjects, and genetic data), and the outcome results separated by individual genotypes. All eligible studies were assessed for methodological quality by KY and VY using the Strengthening the Reporting of Genetic Association (STREGA) study quality score system (Little et al., 2009). The studies were categorized as high (>70%), moderate (50%–70%), or low (<50%) qualities based on the percentages of STREGA adherence, and this was calculated from the quantitative scoring method for the Strengthening the Reporting of Observational Studies in Epidemiology Modified (STROBE-M) checklist (Limaye et al., 2018).

### 2.4 Data analysis

A meta-analysis was performed to calculate pooled estimates using the ratio of means (ROM) method (Friedrich et al., 2011) among the studies that reported their outcomes with the same SNP or complete linkage disequilibrium (LD) ones (Howe et al., 2020) (Supplementary Table S3). The pooled estimates were presented as the ratio values of the means along with 95% CI. All the SNPs included in meta-analysis were assessed for their Hardy–Weinberg equilibrium (HWE) using the chi-square test or *p*-values. For the study, *p* > 0.05 indicated that SNPs were in HWE. Heterogeneity was assessed by the Cochran’s Q-test and *I* ^2^ statistic (Higgins and Thompson, 2002; Higgins et al., 2021), and a subgroup analysis stratified by ethnicity was also conducted to explore the confounding introduced by genetic variability between different populations. All the analyses were performed using Review Manager version 5.4.

## 3 Results

**FIGURE 1:**
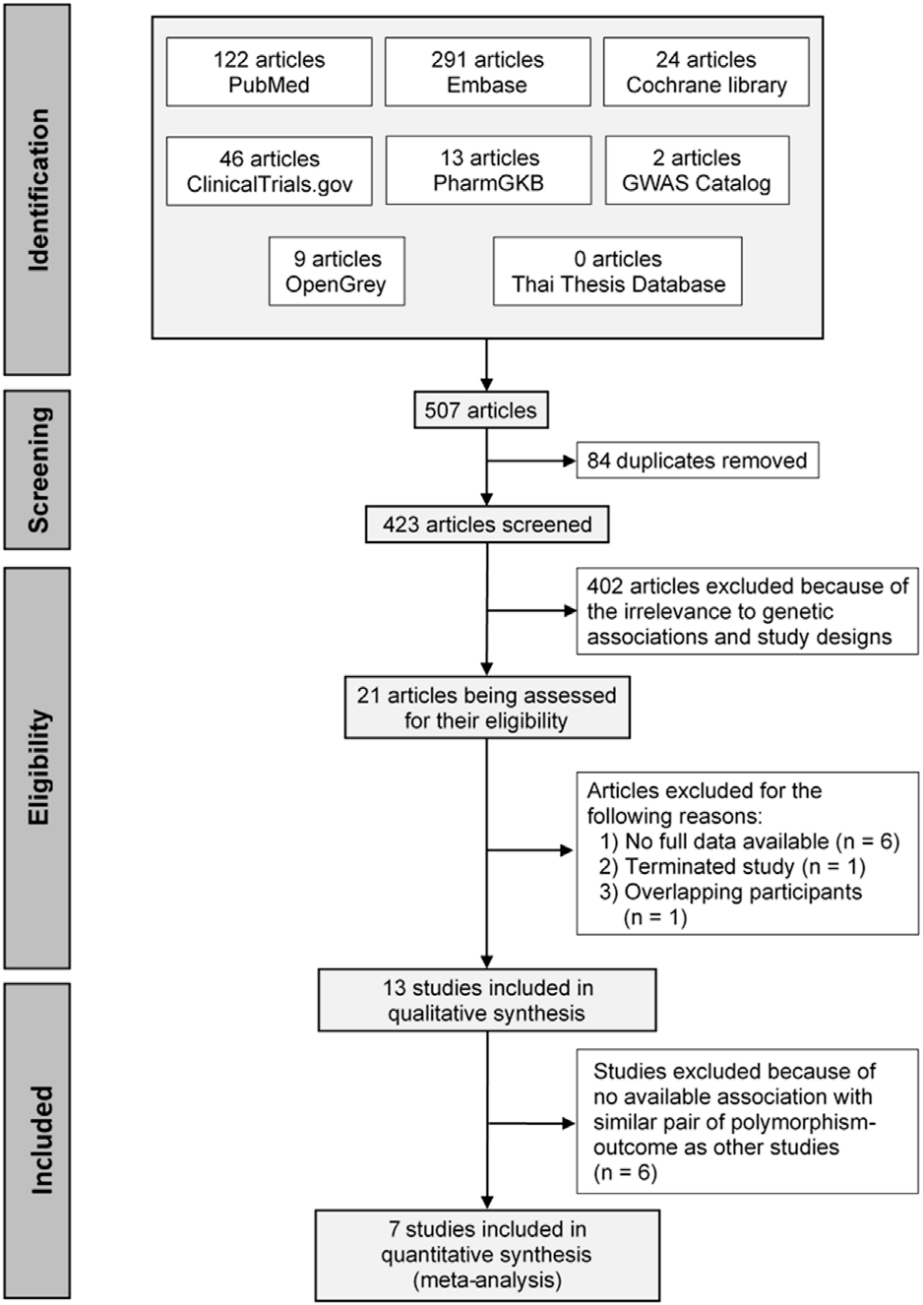
The PRISMA flow diagram describing the study selection process.

A total of 507 articles were initially identified, and 84 duplicates were removed. The remaining articles were screened through the titles and abstracts and refined using the inclusion and exclusion criteria. This resulted in 21 full texts being assessed for eligibility. Of these, 13 studies were included in qualitative synthesis, and seven studies were included in the quantitative synthesis. A PRISMA flow diagram of the process is depicted in Figure 1.

### 3.1 Study characteristics

The characteristics of the 13 included studies are shown in Table 1. Most studies (six studies, study No. 7–11, 13) were conducted in Caucasian adults, and only two studies (study No. 2, 6) were in children. A total of 53 SNPs in 15 genes reported their associations with PK parameters (11 studies, study No. 1–11), efficacy (eight studies, study No. 2, 3, 7–11, 13) and the ADR (2 studies, study No. 2, 12). All studies performed genotyping of candidate polymorphisms involving drug transporters (*ABCC2* and *ABCG2* (study No. 1–3, 5, 7, 9–12)), drug-metabolizing enzymes (*UGT1A1* (study No. 1–5, 7, 9–12), *UGT1A3* (study No. 1–3, 5, 7, 9–12), *UGT1A7* (study No. 12), *UGT1A9* (study No. 12), *CYP1A1* (study No. 2, 5, 7, 9–11), *CYP1A2* and *CYP2D6* (study No. 2, 7, 9–11)), vitamin D pathways (*VDBP*, *VDR*, *CYP24A1*, and *CYP27B1*(study No. 6, 8, 10–11)), and iron metabolic pathways (*HFE* and *TF* (study No. 13)). Six studies (study No. 3, 4, 7, 11–13) were excluded because of no available association with similar pair of polymorphism-outcome as other studies. Finally, a total of 14 SNPs in eight genes involving 367 participants from seven studies (study No. 1, 2, 5, 6, 8–10) were included in a meta-analysis; of these, two studies (study No. 1, 5) were conducted in healthy Chinese subjects and five studies (study No. 2, 6, 8–10) were in β-thalassemia patients; most of them were Caucasian. The characteristics and HWE of the SNPs are shown in Supplementary Table S4.

**TABLE 1:** Characteristics of 13 included studies for qualitative analysis.

Study no.	First author (year)	Country	Study design	Patient characteristic							Measured outcome		STREGA, %
				Ethnic	Subject	Age, year	Male, %	DFX dose, mg/kg/day	Sample size	Reported genes	PK parameter	Others	
1	Cao et al. (2020)	China	Cohort	Chinese	Healthy subjects	26 (22–28)^a^	65.79	20 (single dose)	38	*ABCC2*, *ABCG2*, *UGT1A1*, *UGT1A3*	t_1/2_, T_max_, C_max_, AUC_0–72h_, AUC_0-inf_, Vz/F, CL/F, MRT	-	73.2	
2	Allegra et al. (2017)	Italy	Cohort	Caucasian (95%) Others (5%)	β-thal patients	6.35 (3.33–16.53)^a^	65	25.5 (7.35)^a^	20	*ABCC2*, *ABCG2*, *UGT1A1*, *UGT1A3*, *CYP1A1*, *CYP1A2*, *CYP2D6*	t_1/2_, T_max_, C_max_, C_trough,_ AUC_0–24h_, Vd	*Efficacy*: SF, LIC, *ADR:* SCr, AST, ALT, GGT	73.3	
3	Chirnomas et al. (2009)	US	Cohort	*Adequate responders (AR)* Asian (20%) Black (20%) White (60%) *Inadequate responders (IR)* Asian (40%) Black (10%) White (50%)	Thal and SCD patients	*AR* [9–38]^c^ IR [3–36]^c^	*AR* 60 *IR* 70	35	*AR* 5 *IR* 10	*ABCC2*, *ABCG2*, *UGT1A1*, *UGT1A3*	t_1/2_, AUC_0–24h_, Vd/F, CL/F	Response	56.1	
4	Mattioli et al. (2015)	Italy	Cohort	NA	Thal, MDS, and micro-drepanocytosis patients	31 ± 17^b^ 5–82]^c^	30	25.8 (20.0–32.6)^a^	80	*UGT1A1*	C_ss_	-	69.2	
5	Chen et al. (2020)	China	Cohort	Chinese	Healthy subjects	28.3 ± 6.8^b^ [18–45]^c^	71.4	20 (single dose)	27	*ABCC2*, *ABCG2*, *UGT1A1*, *UGT1A3*, *CYP1A1*	t_1/2_, C_max_, AUC_0–72h_	-	69.0	
6	Allegra et al. (2018a)	Italy	Cohort	Caucasian	β-thal patients	*All patients* 9.00 (3.00–16.00)^a^ *AUC studies* 8.00 (3.00–16.00)^a^	*All patients* 44.4 *AUC studies* 55.6	*All patients* 26.00 (10.38–33.33)^a^ *AUC studies* 26.00 (16.00–33.00)^a^	*All patients* 18 *AUC studies* 9	*CYP24A1*, *CYP27B1*, *VDR*, *VDBP*	t_1/2_, T_max_, C_max_, C_min_, C_trough,_ AUC_0–24h_, Vd	-	73.8	
7	Cusato et al. (2015)	Italy	Cohort	Caucasian (98.2%)	β-thal patients	34.21 (25.11–37.24)^a^	50.9	29.62 (21.93–30.53)^a^	54	*ABCC2*, *ABCG2*, *UGT1A1*, *UGT1A3*, *CYP1A1*, *CYP1A2*, *CYP2D6*	C_trough SS_	Response	68.1	
8	Allegra et al. (2018b)	Italy	Cohort	*All patients* Caucasian (93.9%) Others (6.1%) *AUC studies* Caucasian (94.8%) Others (5.2%)	β-thal patients	34 (18–53)^a^	*All patients* 53.5 *AUC studies* 58.6	29 (13.61–40)^a^	*All patients* 99 *AUC studies* 58	*CYP24A1*, *CYP27B1*, *VDR*, *VDBP*	t_1/2_, T_max_, C_max_, C_min_, C_trough,_ C_trough cutoff_, AUC_0–24h_, AUC_cutoff_,Vd	Response	70.5	
9	Cusato et al. (2016)	Italy	Cohort	Caucasian (98.3%) Others (1.7%)	β-thal patients	33.15 (27.38–36.29)^a^	55	1500 (1218.75–1875)^a,d^	60	*ABCC2*, *ABCG2*, *UGT1A1*, *UGT1A3*, *CYP1A1*, *CYP1A2*, *CYP2D6*	t_1/2_, T_max_, C_max_, AUC_0–24h_, AUC_cutoff,_ Vd	Response	72.7	
10	Allegra et al. (2019)	Italy	Cohort	Caucasian (88.6%) Others (11.4%)	β-thal patients	37.00 (27.50–40.00)^a^	55.2	29.00 (21.96–30.88)^a^	105	*ABCC2*, *ABCG2*, *UGT1A1*, *UGT1A3*, *CYP1A1*, *CYP1A2*, *CYP2D6*, *CYP24A1*, *CYP27B1*, *VDR*, *VDBP*	C_trough_	*Efficacy*: SF, liver stiffness, hepatic T2*, Response	71.4	
11	Allegra et al. (2018c)	Italy	Cohort	White (88.6%)	β-thal patients	37.00 (27.50–40.00)^a^	55.2	29.00 (21.96–30.88)^a^	105	*ABCC2*, *ABCG2*, *UGT1A1*, *UGT1A3*, *CYP1A1*, *CYP1A2*, *CYP2D6*, *CYP24A1*, *CYP27B1*, *VDR*, *VDBP*	C_trough_	*Efficacy*: cardiac T2* Response	70.7	
12	Lee et al. (2013)	Korea	Retro-spective	NA	Patients with IOL	9.0^e^ (1.0–23.0)	64.3	29.4^e^ (17.9–34.1)	98	*ABCC2*, *ABCG2*, *UGT1A1*, *UGT1A3*, *UGT1A7*, *UGT1A9*	-	*ADR*: Hepato-toxicity, Creatinine elevation	79.1	
13	Renda et al. (2014)	Italy	Cohort	Italian	β-thal and SCD patients	37.5 ± 10.6^b^	30.8	NA	13^f^	*HFE*, *TF*	-	*Efficacy*: SF	46.3	

β-thal, β-thalassemia; DFX, deferasirox; *HFE*, high Fe; HH, hereditary hemochromatosis; IOL, iron overload; LIC, Liver iron concentration; MDS, myelodysplastic syndromes; NA, not available; SCr, Serum creatinine; SCD, sickle cell disease; SF, serum ferritin; STREGA, strengthening the reporting of genetic association study; TDA, transfusion-dependent anemia; Thal, thalassemia; *TF*, transferrin; *VDBP*, vitamin D receptor binding protein; *VDR*, vitamin D receptor. AUC_0–24h_, area under the plasma concentration–time curve from 0 to 24 h; AUC_0–72h_, area under the plasma concentration–time curve from 0 to 72 h; AUC_0-inf_, area under the plasma concentration–time curve from 0 to infinity; AUC, _cutoff_, area under the plasma concentration–time curve cutoff; C_max_, maximum concentration, C_min_, minimum concentration, C_trough_, trough concentration, C_ss_, steady-state concentration; C_trough_ ss, steady state trough concentration; C_trough_ _cutoff_, trough concentration cutoff; CL/F, apparent oral clearance; t_1/2_, half-life; MRT, mean residence time; T_max_, time to reach maximum concentration; Vd, volume of distribution; Vd/F, volume of distribution/bioavailability, Vz/F, apparent volume of distribution;

^a^ Median (IQR);

^b^ (mean ± SD);

^c^ [range];

^d^ mg/day;

^e^ Median;

^f^ DFX, monotherapy group.

### 3.2 Quality assessment

The results of the quality assessment are presented in Supplementary Table S5. Most of the studies (12 of 13) yielded STREGA scores above 50%, indicating moderate (4 studies (Chirnomas et al., 2009; Cusato et al., 2015; Mattioli et al., 2015; Chen et al., 2020)) to high quality studies (8 studies (Lee et al., 2013; Cusato et al., 2016; Allegra et al., 2017; Allegra et al., 2018a; Allegra et al., 2018b; Allegra et al., 2018c; Allegra et al., 2019; Cao et al., 2020)).

### 3.3 Previously reported significant genetic associations

Among the 13 studies, 27 genetic polymorphisms in 11 genes were found in 11 studies to be significantly associated with DFX outcomes (Supplementary Table S6). In brief, 24, 15, and seven SNPs showed significant associations with PK parameters, iron-chelating efficacy, and ADRs, respectively. Overall, 15 SNPs influenced more than one type of outcome. For PK parameters, AUC showed the highest number of associations with 12 SNPs in seven genes, followed by a trough concentration (C_trough_) and half-life (8 SNPs in six genes, each parameter). The other two studies by Chirnomas et al. (2009) and Renda et al. (2014) did not find significant genetic associations with DFX outcomes.

#### 3.3.1 Improved pharmacokinetic profile and drug efficacy

The increased DFX exposures were related to four SNPs, including *ABCC2* rs2273697, *UGT1A3* rs3806596, *ABCG2* rs13120400, and *VDR* rs10735810 (Cusato et al., 2015; Cusato et al., 2016; Allegra et al., 2017; Allegra et al., 2018a). Noting that patients bearing *ABCC2* rs2273697 GA and *UGT1A3* rs3806596 GG had higher AUC (Cusato et al., 2016; Allegra et al., 2017) corresponding to higher C_trough_ (Cusato et al., 2015) and C_max_ (Cusato et al., 2016), respectively, and lower Vd (Cusato et al., 2016; Allegra et al., 2017). Moreover, the *UGT1A3* rs3806596 GG was associated with a lower SF level and proposed as a positive predictive factor for DFX effectiveness (AUC >360 μg⋅h/mL) (Cusato et al., 2016). The *ABCG2* rs13120400 CC was also able to predict the efficient AUC in adult β-thalassemia patients (Cusato et al., 2016), whereas it was associated with higher cardiac iron level (Allegra et al., 2018c). The *VDR* rs10735810 CC was found to be a positive predictor of AUC and C_max_, and related to the decreased Vd and half-life in children with β-thalassemia (Allegra et al., 2018a).

#### 3.3.2 Reduced pharmacokinetic profile and drug efficacy

On the other hand, the reduced DFX dispositions were reported in 10 SNPs including *ABCC2* rs717620, *UGT1A1* rs887829, *UGT1A3* rs1983023, *CYP24A1* rs2248359 and rs2585428, *CYP27B1* rs4646536 and rs10877012, *VDR* rs7975232 and rs11568820, and *VDBP* rs7041 (Cusato et al., 2015; Cusato et al., 2016; Allegra et al., 2018a; Allegra et al., 2018b; Cao et al., 2020; Chen et al., 2020). Chinese carrying the T allele of *ABCC2* rs717620 had lower DFX AUC and clearance concurring with shorter half-life and mean residence time (MRT) (Cao et al., 2020). The Chinese T carriers of *UGT1A1* rs887829 (UGT1A1*80) had lower AUC and half-life (Chen et al., 2020). However, this SNP showed opposite associations in other Caucasian-majority populations, that is, longer half-life (Cusato et al., 2016), higher C_trough_ (Cusato et al., 2015), lower SF level, and normal liver iron concentrations (Allegra et al., 2017; Allegra et al., 2019). The associations between *UGT1A3* rs1983023 and decreased levels of AUC, C_max_, half-life, but lower LS level were found in patients with β-thalassemia (Cusato et al., 2016). Furthermore, *CYP24A1* rs2248359 TC/CC and rs2585428 GG were associated with a decreased DFX AUC in pediatric (Allegra et al., 2018a) and adult patients (Allegra et al., 2018b), respectively. These 2 SNPs also demonstrated associations with inferior outcomes, including several PK parameters and cardiac T2* values, reflecting high cardiac iron levels (Allegra et al., 2018a; Allegra et al., 2018b). In addition, decreased minimum concentration (C_min_) was reported in patients bearing the C allele of rs4646536 and the T allele of rs10877012 in *CYP27B1*. The latter was also associated with higher LS value (Allegra et al., 2018a). Among polymorphisms involved with vitamin D pathways, *VDR* rs7975232 AA and G allele carriers of *VDR* rs11568820 and *VDBP* rs7041 were negative predictors of DFX AUC (and other PK parameters) (Allegra et al., 2018a; Allegra et al., 2018b), whereas other SNPs remain controversial. *VDBP* rs7041 was also associated with higher LS values, suggesting reduced efficacy. Apart from the SNPs mentioned above, *ABCG2* rs2231142 GA was associated with lower cardiac T2* values, suggesting lower cardiac iron-chelating efficacy (Allegra et al., 2018c).

#### 3.3.3 Drug toxicity outcomes

With respect to adverse effects, three SNPs were associated with increased serum creatinine, assuming higher renal toxicity. These included *UGT1A1* rs4148323 AA (UGT1A1*6) (Lee et al., 2013), *CYP1A1* rs2606345 AA and rs4646903 TC/CC (Allegra et al., 2017). Patients with *ABCC2* rs717620 and/or rs369192412 were at increased risk of developing hepatotoxicity (Lee et al., 2013). Conversely, *CYP1A2* rs762551 AC/CC and *UGT1A3* rs1983023 TT were associated with lower serum creatinine levels and lower levels of gamma-glutamyltransferase (GGT) enzyme, respectively (Allegra et al., 2017).

### 3.4 Meta-analysis

The associations between genetic polymorphisms and DFX outcomes were evaluated in seven studies (study No. 1, 2, 5, 6, 8–10, Supplementary Table S7). They included seven SNPs with C_min_ (study No. 6, 8) and half-life (study No. 1, 2, 5, 6, 8, 9), five SNPs with AUC (study No. 1, 2, 5, 6, 8, 9), four SNPs with C_max_ (study No. 1, 2, 5, 6, 8, 9), two SNPs with C_trough_ (study No. 6, 10) and Vd (study No. 2, 9), and one SNP with T_max_ (study No. 1, 2, 9) and SF level (study No. 2, 10)).

**FIGURE 2:**
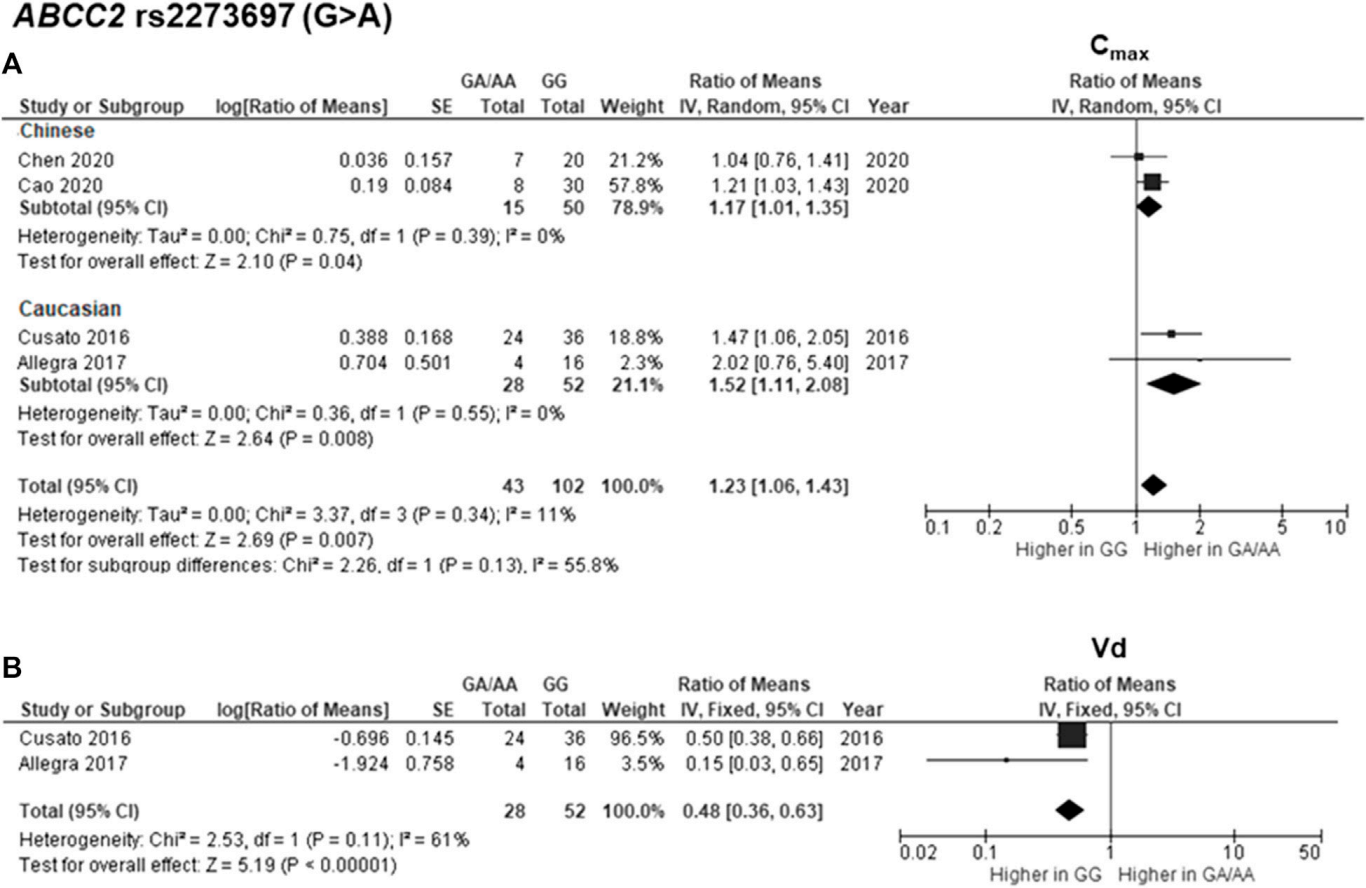
Forest plots for associations between *ABCC2* rs2273697 (GA/AA vs. GG genotypes) and **(A)** maximum concentration (C_max_) with subgroup analysis by ethnicity or **(B)** volume of distribution (Vd) of deferasirox.

**FIGURE 3:**
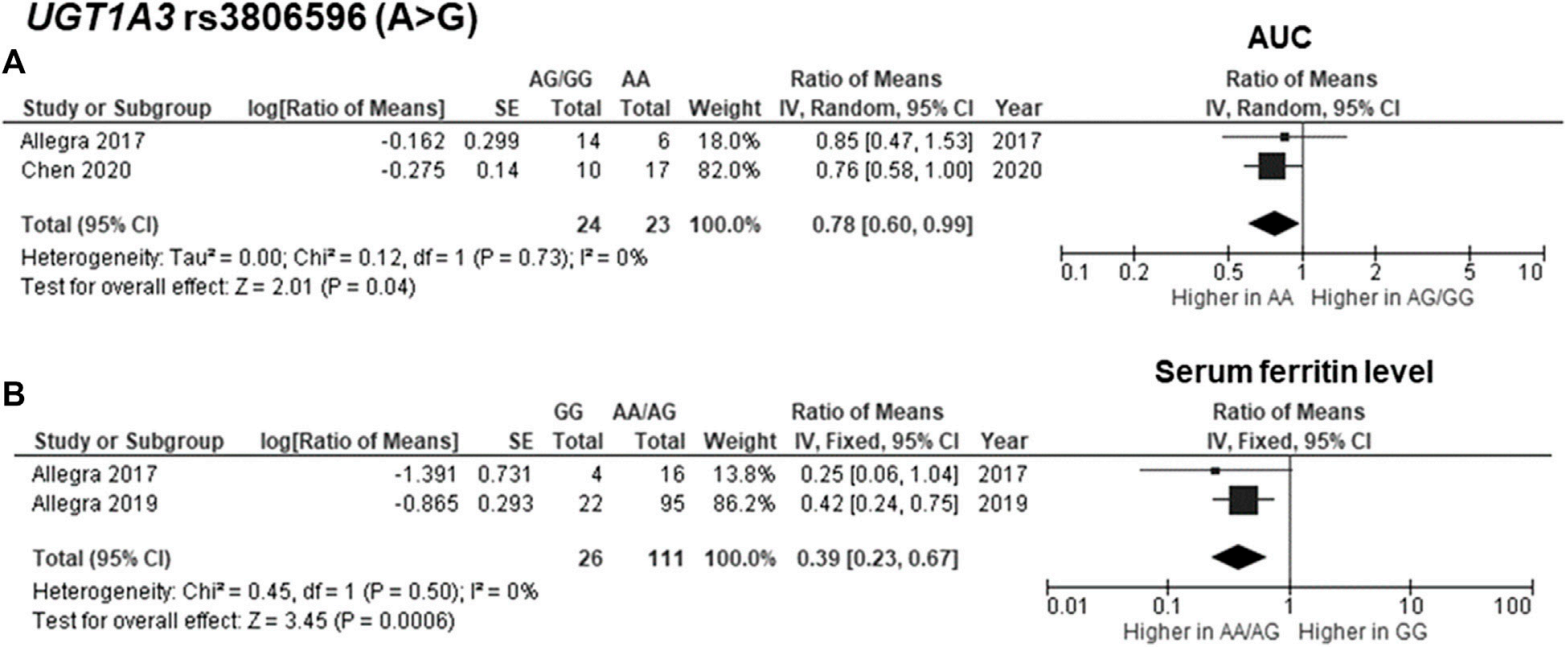
Forest plots for associations between *UGT1A3* rs3806596 (GG vs. AA/AG genotypes) and **(A)** area under the curve (AUC) of deferasirox or **(B)** serum ferritin level.

**FIGURE 4:**
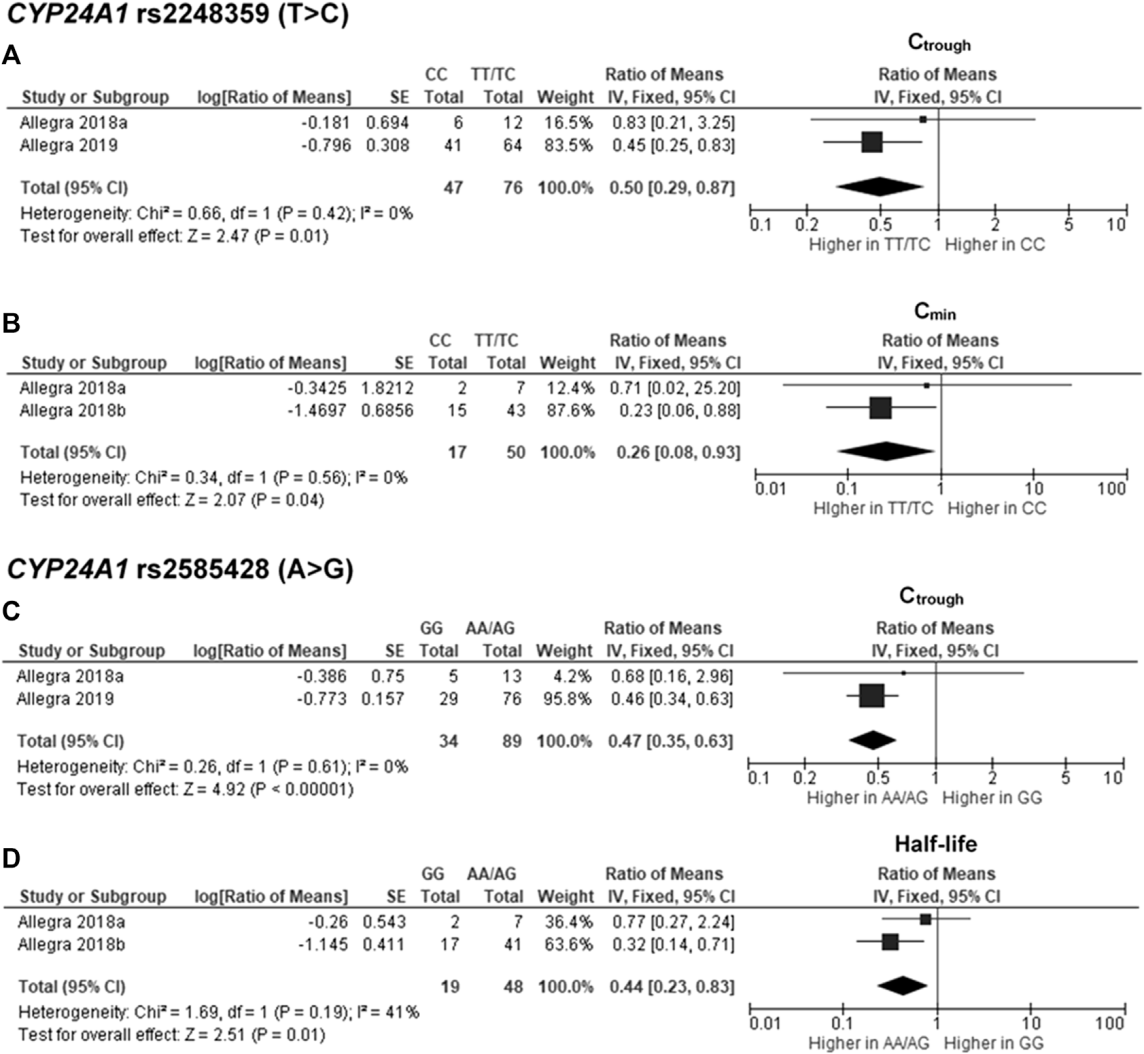
Forest plots for associations between *CYP24A1* rs2248359 (CC vs. TT/TC genotypes) and **(A)** trough concentration (C_trough_) or **(B)** minimum concentration (C_min_); *CYP24A1* rs2585428 (GG vs. AA/AG genotypes) and **(C)** C_trough_ or **(D)** half-life of deferasirox.

The findings indicated significant associations between four genetic polymorphisms in *ABCC2*, *UGT1A3*, and *CYP24A1* and the PK parameters. Subjects carrying the A allele (AG/AA genotypes) of *ABCC2* rs2273697 had a 1.23-fold increase in C_max_ compared with those carrying the GG genotype (95% CI: 1.06–1.43; *p* = 0.007, *I* ^2^ = 11%) (Figure 2A). Moreover, the subgroup analysis according to ethnicity demonstrated a significant association for the Chinese (ROM 1.17, 95% CI: 1.01–1.35; *p* = 0.04, *I* ^2^ = 0%) and Caucasian (ROM 1.52, 95% CI: 1.11–2.08; *p* = 0.008, *I* ^2^ = 0%) subcategories. Besides, the A allele carriers of rs2273697 had a 2.08-fold decreased Vd compared to those with GG genotype (ROM = 0.48; 95% CI: 0.36–0.63; *p* < 0.00001, *I* ^2^ = 61%) (Figure 2B). Regarding drug metabolism, a significant attenuated AUC was observed in subjects with *UGT1A3* rs3806596 AG/GG genotypes by 1.28-fold (ROM 0.78; 95% CI: 0.60–0.99; *p* = 0.04, *I* ^2^ = 0%) (Figure 3A). Similarly, patients carrying homozygous variant (GG genotype) of *UGT1A3* rs3806596 had a significantly lower level of SF compared to A allele carriers (ROM 0.39; 95% CI: 0.23–0.67; *p* = 0.0006, *I* ^2^ = 0%) (Figure 3B). Moreover, two SNPs of *CYP24A1* were associated with PK parameters. The rs2248359 CC and rs2585428 GG genotypes were significantly associated with decreased C_trough_ by two-fold (ROM 0.50; 95% CI: 0.29–0.87; *p* = 0.01, *I* ^2^ = 0%) and 2.13-fold (ROM 0.47; 95% CI: 0.35–0.63; *p* < 0.00001, *I* ^2^ = 0%), respectively (Figures 4A,C). The rs2248359 CC also showed a significant association with a decreased C_min_ by 3.85-fold (ROM 0.26; 95% CI: 0.08–0.93; *p* = 0.04, *I* ^2^ = 0%) (Figure 4B). Consistently, rs2585428 GG was significantly associated with a shorter half-life by 2.27-fold (ROM 0.44; 95% CI: 0.23–0.83; *p* = 0.01, *I* ^2^ = 41%) (Figure 4D). In addition, from the six groups of LD SNPs (A, B, C, D, E, and F), only two (C and D) were pooled in the meta-analyses of AUC (study No. 1, 2, 5 and 9) and C_max_ (study No. 1, 2, 5 and 9) (Supplementary Table S3). All the meta-analysis results are presented in Supplementary Table S7.

## 4 Discussion

To our knowledge, this is the first study to comprehensively evaluate the influence of genetic polymorphisms on clinical and pharmacological responses of DFX using a systematic review and meta-analysis approach. All the included studies investigated candidate polymorphisms in genes with known functions related to DFX pharmacokinetics/pharmacodynamics and thalassemia disease. All meta-analysis results demonstrated no observed heterogeneity. Our findings revealed the significant association between *ABCC2* rs2273697 the increased C_max_ of DFX, supporting the previously reported associations with other PK outcomes (Cusato et al., 2015; Allegra et al., 2017). This meta-anaysis confirmed the association between rs2273697 and the lower Vd of DFX which was described by Cusato et al., 2016 and Allegra et al., 2017. The previous studies also reported associations between rs2273697 and the toxicity of methotrexate, which is eliminated via the ABCC2 (MRP2) transporter (Izzedine et al., 2006; Ranganathan et al., 2008). Conversely, *ABCC2* rs717620 showed associations with PK parameters leading to inadequate response (Cao et al., 2020). Indeed, *in vitro* assays demonstrated the opposite functions of these missense variants, supporting our finding, that is, rs2273697 (1249G>A) decreased transport activity (Wen et al., 2017) but rs717620 (−24C>T) increased promotor function of *ABCC2*, which could result in higher expression of this transporter (Nguyen et al., 2013).

Regarding the major metabolism pathway of DFX (Waldmeier et al., 2010), several polymorphisms in UGTs revealed significant associations with PK parameters, efficacy, and toxicity outcomes. However, some variants in this gene demonstrated inconsistent clinical associations between different populations or outcomes. UGT1A1*6 and *28, missense variants leading to reduced enzyme activity (Beutler et al., 1998), showed clinical significance as pathogenic variants for a hyperbilirubinemia condition called Gilbert’s syndrome (Landrum et al., 2017), which is associated with irinotecan toxicity in Asians (Han et al., 2014; Atasilp et al., 2016). For DFX, UGT1A1*6 was a risk factor of creatinine elevation indicating renal toxicity (Lee et al., 2013); but UGT1A1*28 influenced the decrease in steady-state DFX concentrations (Mattioli et al., 2015). In addition, UGT1A1*80, an intron variant in a strong LD with UGT1A1*28 (Gammal et al., 2016), was associated with the improved PK profile and efficacy outcomes in Caucasian (Cusato et al., 2015; Cusato et al., 2016; Allegra et al., 2017; Allegra et al., 2019), but with the reduced PK profile in Chinese (Chen et al., 2020). It should be noted that the distribution of this variant in the Chinese population reported in Chen et al., in 2020 was not in HWE. Another possibility is that a single SNP might not explain all the effects. Due to different genetic variability and distribution across populations, one SNP may be a part of different haplotypes leading to distinct phenotypes and outcomes (Sai et al., 2004). Interestingly, our meta-analysis revealed that rs3806596 (A > G), a non-coding variant in *UGT1A3* (Howe et al., 2020), was associated with reduced DFX AUC, as well as improved efficacy by lowering SF levels. These associations could not be directly compared due to the difference in the grouping of genotypes (AG/GG vs. AA for AUC and GG vs. AA/AG for SF level). In addition, a significant association between rs3806596 and increased AUC was found in Caucasian thalassemia patients (Cusato et al., 2016), whereas the meta-analysis included a population combining Chinese healthy adults (Chen et al., 2020) with Caucasian pediatrics (Allegra et al., 2017). Previous research found that hepatic glucuronidation activity in children was lower than in adults (Strassburg et al., 2002), indicating less dependence on UGTs for DFX metabolism. Moreover, the pathology of thalassemia may affect the pharmacokinetic of drugs metabolized by glucuronidation (Temsakulphong et al., 2004). Due to various confounding factors that could affect glucuronidation, as mentioned above, we suggested further research to study the genetic associations between UGT variants and DFX responses in a specific population concerning these factors.

As the relationship between vitamin D deficiency and iron deficiency or β-thalassemia has been indicated (Napoli et al., 2006; Mogire et al., 2022), variants in genes related to vitamin D were investigated for their impact on DFX responses. Among these, two variants in *CYP24A1* showed associations with the reduced PK parameters in adult and pediatric patients with β-thalassemia. The meta-analysis confirmed the previously reported associations of rs2248359 with C_trough_ and C_min_, and of rs2585428 with C_trough_ and the half-life of DFX (Allegra et al., 2018b). Additionally, rs2585428 was associated with a lower value of cardiac T2*, indicating the inferior efficacy of DFX in removing cardiac iron. (Allegra et al., 2018c). Furthermore, rs2248359 is located in the transcription factor binding site in the promotor region, and rs2585428 is in the intron of *CYP24A1* (Howe et al., 2020). To date, there has been no reports on the direct function of these two variants. It is interesting to further investigate the function of these variants and validate their associations in different ethnic groups. Furthermore, rs4646536 (C allele) and rs10877012 (T allele) in *CYP27B1* significantly decreased PK and the efficacy of DFX (Allegra et al., 2018a; Allegra et al., 2019). These two intron variants were in complete LD and exhibited a similar function to increase 1α-hydroxylase, a gene product of *CYP27B1* involved with vitamin D metabolism (Clifton-Bligh et al., 2011). Interestingly, the 1000 Genomes Project reported the disparity of the frequencies of rs4646536 (C allele) and rs10877012 (T allele) among different ethnicities (Auton et al., 2015), that is, 32% in Europeans but 64%–65% in East Asians. These results suggest the significance of variations in genes involved with vitamin D metabolism, especially in East Asian populations.

This study has some limitations. First, due to the different genotype grouping in previous studies, some significant associations could not be pooled in our meta-analysis. Second, the polygenic effect on drug responses could not be analyzed because we could not access to the individual data in previous publications. Although the mechanism underlying DFX as iron chelation is simple, the target organs in which iron is chelated include the bloodstream and the liver and heart (Porter and Viprakasit, 2014). DFX pharmacokinetics is also complicated, including its metabolism via several pathways and enterohepatic circulation (Waldmeier et al., 2010). In addition, personal conditions, such as age, underlying disease, physiological alteration, and organ impairment, may affect drug properties (Whittaker et al., 2018; García-Cortés and García-García, 2022), drug efficacy and toxicity (Veyssier, 1992). Therefore, the response to DFX can be influenced by various genetic polymorphisms involving pathophysiology and disease progression. These cause difficulties in the interpretation and evaluation of the consequences of genetic variants on DFX PK, efficacy, and toxicity outcomes.

## 5 Conclusion

Polymorphisms in *ABCC2* should be considered for both increasing and decreasing DFX responses. The impacts of *ABCC2* rs2273697 and rs71762 on DFX responses and transporter function have been well established by clinical and *in vitro* studies, respectively, and should be evaluated for clinical use. We recommend validating the controversial associations through clinical studies (e.g., polymorphisms in UGT genes) and *in vitro* methods (e.g., *CYP24A1* rs2248359 and rs2585428). These associations need to be further clarified in terms of ethnic differences that may possibly affect allele frequency, the physiological difference between children *versus* adults or between healthy persons *versus* patients, and the pharmacological mechanism by which genetic variation influences DFX response. Furthermore, to integrate the total influence of functional genetic polymorphisms, polygenic association analysis should be performed and ultimately used to predict drug responses.

## Data Availability

The original contributions presented in the study are included in the article/Supplementary Material, further inquiries can be directed to the corresponding authors.
