# Supplementary material for: Genetic polymorphisms influencing deferasirox pharmacokinetics, efficacy, and adverse drug reactions: a systematic review and meta-analysis
Source: Front Pharmacol. 2023 May 16;14:1069854. doi: 10.3389/fphar.2023.1069854 (PMC10227503; doi:10.3389/fphar.2023.1069854)
Supplement: Supplementary file 1 [file DataSheet1.pdf]

Supplementary Material

Supplementary Material

1 Supplementary Tables

Supplementary Table S1 Search terms and search strategies.

Database	Search terms and search strategies	Number of articles found
Pubmed	(Deferasirox) OR (ICL?670) OR (ICL?670A) OR (Exjade)	1,192
	(Pharmacogenetic*) OR (Genetic*) OR (Genetic) OR ("Personalized medicine") OR ("Precision medicine") OR (Polymorphism*) OR (Polymorphism) OR (SNP) OR (SNPs) OR ("Genetic Variation*") OR ("Genetic Variations")	4,691,737
	(Pharmacokinetic*) OR (Pharmacokinetics) OR (Response*) OR (Response) OR (Efficacy) OR (Effectiveness) OR ("Treatment Outcome") OR (Adverse*) OR (Adverse) OR (Toxic*) OR ("side effect*") OR ("side effects")	13,235,775
	((Deferasirox) OR (ICL?670) OR (ICL?670A) OR (Exjade)) AND ((Pharmacogenetic*) OR (Genetic*) OR (Genetic) OR ("Personalized medicine") OR ("Precision medicine") OR (Polymorphism*) OR (Polymorphism) OR (SNP) OR (SNPs) OR ("Genetic Variation*") OR ("Genetic Variations")) AND ((Pharmacokinetic*) OR (Pharmacokinetics) OR (Response*) OR (Response) OR (Efficacy) OR (Effectiveness) OR ("Treatment Outcome") OR (Adverse*) OR (Adverse) OR (Toxic*) OR ("side effect*") OR ("side effects"))	123
EMBASE	(Deferasirox) OR (ICL670*) OR ("ICL 670*") OR (Exjade)	4,053
	(Pharmacogenetic*) OR (Genetic*) OR (Genetic) OR ("Personalized medicine") OR ("Precision medicine") OR (Polymorphism*) OR (Polymorphism) OR (SNP) OR (SNPs) OR ("Genetic Variation*") OR ("Genetic Variations")	3,853,039
	(Pharmacokinetic*) OR (Pharmacokinetics) OR (Response*) OR (Response) OR (Efficacy) OR (Effectiveness) OR ("Treatment Outcome") OR (Adverse*) OR (Adverse) OR (Toxic*) OR ("side effect*") OR ("side effects")	10,308,956
	((Deferasirox) OR (ICL670*) OR ("ICL 670*") OR (Exjade)) AND ((Pharmacogenetic*) OR (Genetic*) OR (Genetic) OR ("Personalized medicine") OR ("Precision medicine") OR (Polymorphism*) OR (Polymorphism) OR (SNP) OR (SNPs) OR ("Genetic Variation*") OR ("Genetic Variations")) AND ((Pharmacokinetic*) OR (Pharmacokinetics) OR (Response*) OR (Response) OR (Efficacy) OR (Effectiveness) OR ("Treatment Outcome") OR (Adverse*) OR (Adverse) OR (Toxic*) OR ("side effect*") OR ("side effects"))	291

Cochrane CENTRAL	(Deferasirox) OR (ICL670*) OR ("ICL 670*") OR (Exjade)	375
	(Pharmacogenetic*) OR (Genetic*) OR (Genetic) OR ("Personalized medicine") OR ("Precision medicine") OR (Polymorphism*) OR (Polymorphism) OR (SNP) OR (SNPs) OR ("Genetic Variation*") OR ("Genetic Variations")	45,664
	(Pharmacokinetic*) OR (Pharmacokinetics) OR (Response*) OR (Response) OR (Efficacy) OR (Effectiveness) OR ("Treatment Outcome") OR (Adverse*) OR (Adverse) OR (Toxic*) OR ("side effect*") OR ("side effects")	971,048
	(Deferasirox) OR (ICL670*) OR ("ICL 670*") OR (Exjade) AND (Pharmacogenetic*) OR (Genetic*) OR (Genetic) OR ("Personalized medicine") OR ("Precision medicine") OR (Polymorphism*) OR (Polymorphism) OR (SNP) OR (SNPs) OR ("Genetic Variation*") OR ("Genetic Variations”) AND (Pharmacokinetic*) OR (Pharmacokinetics) OR (Response*) OR (Response) OR (Efficacy) OR (Effectiveness) OR ("Treatment Outcome") OR (Adverse*) OR (Adverse) OR (Toxic*) OR ("side effect*") OR ("side effects")	24
ClinicalTrials.gov	(Deferasirox)	108
	(Pharmacogenetic) OR (Genetic) OR ("Personalized medicine") OR ("Precision medicine") OR (Polymorphism) OR (SNP) OR (Genetic Variation)	24,591
	(Pharmacokinetic OR Pharmacokinetics) OR (Response) OR (Efficacy) OR (Effectiveness) OR ("Treatment Outcome") OR (Adverse) OR (Toxic) OR ("side effect")	300,495
	(Deferasirox) AND ((Pharmacogenetic) OR (Genetic) OR ("Personalized medicine") OR ("Precision medicine") OR (Polymorphism) OR (SNP) OR (Genetic Variation)) AND (Pharmacokinetic OR Pharmacokinetics) OR (Response) OR (Efficacy) OR (Effectiveness) OR ("Treatment Outcome") OR (Adverse) OR (Toxic) OR ("side effect")	46
PharmGKB	(Deferasirox) OR (ICL670*)	13
GWAS Catalog	(Deferasirox) OR ("ICL 670*")	2
OpenGrey	(Deferasirox) OR (Exjade)	9
Thai Thesis Database	(Deferasirox) OR ("ICL 670") OR (Exjade) OR (เอ็กซ์เจด) OR (ดีเฟอราซิรอก) OR (ดีเฟอราซิร็อกซ์)	0

Supplementary Table S2 Preferred Reporting Items for Systematic Reviews and Meta-Analyses (PRISMA Checklist).

Section/topic	Item No.	Checklist item	Section and topic (page No.)
**Title**			
Title	1	Identify the report as a systematic review, meta-analysis, or both	First page (1)
**Abstract**			
Structured summary	2	Provide a structured summary including, as applicable, background, objectives, data sources, study eligibility criteria, participants, interventions, study appraisal and synthesis methods, results, limitations, conclusions and implications of key findings, systematic review registration number	Abstract (1)
**Introduction**			
Rationale	3	Describe the rationale for the review in the context of what is already known	Introduction (2)
Objectives	4	Provide an explicit statement of questions being addressed with reference to participants, interventions, comparisons, outcomes, and study design (PICOS)	Introduction (2)
**Methods**			
Protocol and registration	5	Indicate if a review protocol exists, if and where it can be accessed (such as web address), and, if available, provide registration information including registration number	Study selection (2)
Eligibility criteria	6	Specify study characteristics (such as PICOS, length of follow-up) and report characteristics (such as years considered, language, publication status) used as criteria for eligibility, giving rationale	Data sources and search strategy (2), and Study selection (2)
Information sources	7	Describe all information sources (such as databases with dates of coverage, contact with study authors to identify additional studies) in the search and date last searched	Data sources and search strategy (2)
Search	8	Present full electronic search strategy for at least one database, including any limits used, such that it could be repeated	Data sources and search strategy (2)
Study selection	9	State the process for selecting studies (that is, screening, eligibility, included in systematic review, and, if applicable, included in the meta-analysis)	Study selection (2)
Data collection process	10	Describe method of data extraction from reports (such as piloted forms, independently, in duplicate) and any processes for obtaining and confirming data from investigators	Data extraction and quality assessment (2)
Data items	11	List and define all variables for which data were sought (such as PICOS, funding sources) and any assumptions and simplifications made	Data sources and search strategy (2)

Section/topic	Item No.	Checklist item	Section and topic (page No.)
Risk of bias in individual studies	12	Describe methods used for assessing risk of bias of individual studies (including specification of whether this was done at the study or outcome level), and how this information is to be used in any data synthesis	Data extraction and quality assessment (2)
Summary measures	13	State the principal summary measures (such as risk ratio, difference in means).	Data analysis (2-3)
Synthesis of results	14	Describe the methods of handling data and combining results of studies, if done, including measures of consistency (such as I^2 statistic) for each meta-analysis	Data analysis (2-3)
Risk of bias across studies	15	Specify any assessment of risk of bias that may affect the cumulative evidence (such as publication bias, selective reporting within studies)	Data sources and search strategy (2), Data analysis (2-3)
Additional analyses	16	Describe methods of additional analyses (such as sensitivity or subgroup analyses, meta-regression), if done, indicating which were pre-specified	Data analysis (2-3)
**Results**			
Study selection	17	Give numbers of studies screened, assessed for eligibility, and included in the review, with reasons for exclusions at each stage, ideally with a flow diagram	Results (3)
Study characteristics	18	For each study, present characteristics for which data were extracted (such as study size, PICOS, follow-up period) and provide the citations	Study characteristics (3)
Risk of bias within studies	19	Present data on risk of bias of each study and, if available, any outcome-level assessment (see item 12).	Quality assessment (3)
Results of individual studies	20	For all outcomes considered (benefits or harms), present for each study (a) simple summary data for each intervention group and (b) effect estimates and confidence intervals, ideally with a forest plot	Previously reported significant genetic associations (3-7), Improved pharmacokinetic profile and drug efficacy (4-6), Reduced pharmacokinetic profile and drug efficacy (4-7), Drug toxicity outcomes (4-7)
Synthesis of results	21	Present results of each meta-analysis done, including confidence intervals and measures of consistency	Meta-analysis (7)
Risk of bias across studies	22	Present results of any assessment of risk of bias across studies (see item 15)	Meta-analysis (7)
Additional analysis	23	Give results of additional analyses, if done (such as sensitivity or subgroup analyses, meta-regression) (see item 16)	Meta-analysis (7)

Section/topic	Item No.	Checklist item	Section and topic (page No.)
**Discussion**			
Summary of evidence	24	Summarize the main findings including the strength of evidence for each main outcome; consider their relevance to key groups (such as health care providers, users, and policy makers)	Discussion (7-10)
Limitations	25	Discuss limitations at study and outcome level (such as risk of bias), and at review level (such as incomplete retrieval of identified research, reporting bias)	Discussion (10)
Conclusions	26	Provide a general interpretation of the results in the context of other evidence, and implications for future research	Discussion (10)
**Funding**			
Funding	27	Describe sources of funding for the systematic review and other support (such as supply of data) and role of funders for the systematic review	Funding (10)

Supplementary Table S3 Group of SNPs with completed linkage disequilibrium (LD) identified by D' = 1 of the indicated population.

LD group	Population	Super population	SNPs
A	CHB	EAS	*CYP27B1* rs4646536, *CYP27B1* rs10877012
	JPT	EAS	*CYP27B1* rs4646536, *CYP27B1* rs10877012
	TSI	EUR	*CYP27B1* rs4646536, *CYP27B1* rs10877012
B	CHB	EAS	*UGT1A1* rs4124874, *UGT1A3* rs3755319
	JPT	EAS	*UGT1A1* rs4124874, *UGT1A3* rs3755319
	TSI	EUR	*UGT1A1* rs4124874, *UGT1A3* rs3755319
C	CHB	EAS	*UGT1A3* rs10929302, *UGT1A1* rs887829, *UGT1A3* rs1983023, *UGT1A3* rs6431625
	JPT	EAS	*UGT1A3* rs10929302, *UGT1A1* rs887829, *UGT1A3* rs1983023, *UGT1A3* rs6431625
	TSI	EUR	*UGT1A3* rs10929302, *UGT1A1* rs887829
D	CHB	EAS	*UGT1A3* rs3806596, *UGT1A3* rs3755319, *UGT1A1* rs4124874, *UGT1A3* rs7574296, *UGT1A3* rs6706232, *UGT1A3* rs3821242
	JPT	EAS	*UGT1A3* rs3806596, *UGT1A3* rs3755319, *UGT1A1* rs4124874, *UGT1A3* rs7574296, *UGT1A3* rs6706232, *UGT1A3* rs3821242
	TSI	EUR	*UGT1A3* rs3806596, *UGT1A3* rs3755319, *UGT1A1* rs4124874, *UGT1A3* rs7574296, *UGT1A3* rs6706232, *UGT1A3* rs3821242
E	CHB	EAS	*UGT1A7* rs17868323, *UGT1A7* rs17868324, *UGT1A7* rs17863778
	JPT	EAS	*UGT1A7* rs17868323, *UGT1A7* rs17868324, *UGT1A7* rs17863778
	TSI	EUR	*UGT1A7* rs17868323, *UGT1A7* rs17868324, *UGT1A7* rs17863778
F	CHB	EAS	-
	JPT	EAS	*VDR* rs731236, *VDR* rs1544410
	TSI	EUR	-

CHB, Han Chinese in Beijing, China; EAS, East Asian; EUR, European; JPT, Japanese in Tokyo, Japan; LD, linkage disequilibrium; SNPs, Single nucleotide polymorphisms; TSI, Toscani in Italia.

Supplementary Table S4 Characteristics and Hardy–Weinberg Equilibrium of 14 SNPs included in meta-analysis

Genes	RefSeq (Position)[a]	rs ID	Ref>Alt allele	Consequence	Study No.	Alt AF	HWE *p*-value
ABCC2 (*MRP2*)	NM_000392.5 (c.1249)	rs2273697	G>A	Missense Variant	1	0.11	1.00
					2	0.100[b]	> 0.05
					5	0.129	0.439[c]
					9	0.209	0.209[c]
ABCG2 (*BCRP1*)	NM_004827.3 (c.1194+928)	rs13120400	T>C	Intron Variant	2	NA	> 0.05
					9	0.325	0.697[c]
UGT1A1	NM_000463.2 (c.-364)	rs887829	C>T	Upstream Transcript Variant	2	0.400[b]	> 0.05
					5	0.130	**0.008**[c]
					9	0.342	0.568[c]
UGT1A3	NM_019093.2 (c.-751)	rs1983023	C>T	Upstream Transcript Variant	2	NA	> 0.05
					9	0.583	0.825[c]
	NM_019093.2 (c.-66)	rs3806596	A>G	5 Prime UTR Variant	2	0.450[b]	> 0.05
					5	0.241[f]	0.131[c]
					9	0.484	0.599[c]
					10	0.47	0.2654
CYP1A1	NM_000499.5 (c.-27+606)	rs2606345	C>A	Intron Variant	2	NA	> 0.05
					5	0.093	0.596[c]
CYP24A1	NG_008334.1 (g.3999)	rs2248359	T>C	2KB Upstream Variant	6[d]	0.611	0.9277
					6[e]	0.500	1.000
					8[d]	0.394	0.3406
					8[e]	0.388	0.6182
					10	0.397	0.5906
	NG_008334.1 (g.8620)	rs2585428	A>G	Intron Variant	6[d]	0.472[f]	0.5572
					6[e]	0.389[f]	0.6968
					8[d]	0.444	0.4309

					8[e]	0.457	1
					10	0.461	0.6947
	NG_008334.1 (g.22776)	rs927650	C>T	Intron Variant	6[d]	0.556[g]	0.9435
					8[d]	0.490	1
CYP27B1	NM_000785.3 (c.-1261)	rs10877012	G>T	2KB Upstream Variant	6[d]	0.083	1.000
					8[d]	0.258	0.2846
	CYP27B1 c.+2838 NM_000785.3 (c.1137-29)	rs4646536	C>T	Intron Variant	6[d]	0.083	1.000
					8[d]	0.273	0.1038
VDR	Bsml NM_000376.2 (c.1024+283)	rs1544410	G>A	Intron Variant	6[d]	0.389	0.2668
					8[d]	0.429	0.3280
	TaqI NM_000376.2 (c.1056)	rs731236	T >C	Synonymous Variant	6[d]	0.472	1.0000
					8[d]	0.414	0.4947
	ApaI NM_000376.2 (c.1025-49)	rs7975232	C>A	Intron Variant	6[d]	0.333	0.5003
					8[d]	0.460	0.4871

HWE, Hardy–Weinberg Equilibrium; Alt AF, Alternative allele frequency; RefSeq, Reference Sequence; Ref/Alt allele, Reference allele and alternative allele; SNPs, Single nucleotide polymorphisms.

[a] Reference sequences provided by The Single Nucleotide Polymorphism Database (dbSNP) follows Genome Reference Consortium Human Build 37 patch release 13 (GRCh37.p13) assembly: the prefixes NG_ and NM_ refer to gene/genomic region and coding transcript, respectively. Position c. and g. refer to DNA change position on coding DNA reference sequence (based on a protein-coding transcript) or linear genomic reference sequence, respectively; [b] Alt AF was calculated using the genotype data of the study; [c] HWE *p*-value was calculated using the genotype data of the study; [d] Data considered all patients in the study; [e] Data considered a subgroup of patients in the AUC study; [f] Alt AF was reported as the frequency of complementary allele C; [g] Alt AF was reported as the frequency of complementary allele A.

Supplementary Table S5 Quality assessment of 13 included studies according to Strengthening the Reporting of Genetic Association Study (STREGA) Statement.

**STREGA checklist item**		**Study**												
Study ID<br>First author<br>(Year)		1<br>Cao<br>(2020)	2<br>Allegra<br>(2017)	3<br>Chirnomas<br>(2009)	4<br>Mattioli<br>(2015)	5<br>Chen<br>(2020)	6<br>Allegra<br>(2018a)	7<br>Cusato<br>(2015)	8<br>Allegra<br>(2018b)	9<br>Cusato<br>(2016)	10<br>Allegra<br>(2019)	11<br>Allegra<br>(2018c)	12<br>Lee<br>(2013)	13<br>Renda<br>(2014)
Title and abstract														
Title	1a	0	1	1	0	1	1	0	0	0	0	0	1	1
Abstract	1b	1	1	1	1	1	1	1	1	1	1	1	1	1
Introduction														
Background rationale	2	1	1	1	1	1	1	1	1	0	1	1	1	1
Objectives	3	1	1	1	1	1	1	1	1	1	1	1	1	1
STREGA	3x	0	0	0	1	1	1	0	0	0	0	0	1	0
Methods														
Study design	4	1	1	1	1	1	1	1	1	1	1	1	1	1
Setting	5	0	1	1	0	0	1	1	1	1	1	1	0	0
Participants	6a	1	1	1	1	1	1	1	1	1	1	1	1	0
	6b	NA	NA	NA	NA	NA	NA	NA	NA	NA	NA	NA	NA	NA
STREGA	6x	NA	NA	NA	NA	NA	NA	NA	NA	NA	NA	NA	NA	NA
Variables	7a	1	1	1	1	1	1	1	1	1	1	1	1	1
STREGA	7b	1	1	1	1	1	1	1	1	1	1	1	1	1
Data sources/measurement	8a	1	1	1	1	1	1	1	1	1	1	1	1	1
STREGA	8b	1	1	0	0	1	1	1	1	1	1	1	1	1
Bias	9a	1	0	0	0	0	0	0	0	0	0	0	0	0
STREGA	9b	0	0	0	0	0	0	0	0	0	0	0	0	0
Study size	10	0	0	0	0	0	0	0	0	0	0	0	0	0
Quantitative variables	11	1	1	1	1	1	1	1	1	1	1	1	1	0
STREGA	11x	NA	NA	NA	NA	NA	NA	NA	NA	NA	NA	NA	NA	NA
Statistical methods	12a	1	1	1	1	1	1	1	1	1	1	1	1	0
STREGA	12x	1	1	0	1	1	1	1	1	1	1	1	1	0
	12b	0	1	0	1	0	1	0	1	1	1	1	1	0

STREGA checklist item		Study												
Study ID<br>First author<br>(Year)		1<br>Cao<br>(2020)	2<br>Allegra<br>(2017)	3<br>Chirnomas<br>(2009)	4<br>Mattioli<br>(2015)	5<br>Chen<br>(2020)	6<br>Allegra<br>(2018a)	7<br>Cusato<br>(2015)	8<br>Allegra<br>(2018b)	9<br>Cusato<br>(2016)	10<br>Allegra<br>(2019)	11<br>Allegra<br>(2018c)	12<br>Lee<br>(2013)	13<br>Renda<br>(2014)
	12c	NA	0	NA	NA	NA	NA	0	NA	NA	NA	NA	NA	NA
	12d	NA	NA	NA	NA	NA	NA	0	NA	NA	NA	NA	NA	NA
	12e	0	0	0	0	0	0	1	0	1	0	0	0	0
STREGA	12f	1	1	0	0	0	1	1	1	1	1	1	1	0
STREGA	12g	1	NA	NA	NA	NA	NA	NA	NA	NA	NA	NA	1	NA
STREGA	12h	NA	0	0	0	NA	NA	0	0	0	0	0	0	0
STREGA	12i	0	1	0	0	0	0	1	0	1	0	0	0	0
STREGA	12j	0	0	0	0	0	0	0	0	0	0	0	0	0
Results														
Participants	13a	1	1	1	1	1	1	1	1	1	1	1	1	1
STREGA	13x	1	1	0	1	1	1	1	1	1	1	1	1	1
	13b	NA	1	NA	NA	1	1	0	1	1	NA	NA	NA	NA
	13c	NA	0	NA	NA	0	0	0	0	0	NA	NA	NA	NA
Descriptive data	14a	1	1	1	1	1	1	1	1	1	1	1	1	1
STERGA	14x	0	0	0	0	0	0	0	0	0	0	0	1	1
	14b	NA	1	NA	NA	NA	NA	0	NA	NA	NA	NA	NA	NA
	14c	1	1	1	1	1	1	1	1	1	1	1	1	0
Outcome data	15a	1	1	1	1	1	1	1	1	1	1	1	1	1
STREGA	15x	1	1	0	1	1	1	1	1	1	1	1	1	1
Main data	16a	1	1	1	1	1	1	1	1	1	1	1	1	1
	16b	NA	NA	NA	NA	NA	NA	1	1	1	1	NA	1	NA
	16c	NA	NA	NA	NA	NA	NA	NA	NA	NA	NA	NA	NA	NA
STREGA	16d	0	1	0	1	0	1	1	1	1	1	1	1	0
Other analysis	17a	1	1	0	1	1	1	1	1	1	1	1	1	0
STREGA	17b	1	1	1	NA	1	1	1	1	1	1	1	1	0
STREGA	17c	1	0	0	NA	1	0	1	0	0	0	0	1	1
Discussion														
Key results	18	1	1	1	1	1	1	1	1	1	1	1	1	1
Limitations	19	1	1	1	1	0	0	1	1	0	1	1	0	0

STREGA checklist item		Study												
Study ID<br>First author<br>(Year)		1<br>Cao<br>(2020)	2<br>Allegra<br>(2017)	3<br>Chirnomas<br>(2009)	4<br>Mattioli<br>(2015)	5<br>Chen<br>(2020)	6<br>Allegra<br>(2018a)	7<br>Cusato<br>(2015)	8<br>Allegra<br>(2018b)	9<br>Cusato<br>(2016)	10<br>Allegra<br>(2019)	11<br>Allegra<br>(2018c)	12<br>Lee<br>(2013)	13<br>Renda<br>(2014)
Interpretation	20	1	1	1	1	1	1	1	1	1	1	1	1	0
Generalizability	21	1	1	1	1	1	1	1	1	1	1	1	1	1
Other information														
Funding	22	1	0	1	1	1	0	0	0	1	0	0	1	0
**Total score**														
Achieved/ possible<br>(percent)		30/41<br>(73.2)	33/45<br>(73.3)	23/41<br>(56.1)	27/39<br>(69.2)	29/42<br>(69.0)	31/43<br>(73.8)	32/47<br>(68.1)	31/44<br>(70.5)	32/44<br>(72.7)	30/42<br>(71.4)	29/41<br>(70.7)	34/43<br>(79.1)	19/41<br>(46.3)
**Quality level**		high	high	moderate	moderate	moderate	high	moderate	high	high	high	high	high	low

0, scored 0 (no); 1, scored 1 (yes); NA, not applicable

Supplementary Table S6 The previously reported significant genetic associations for qualitative synthesis.

Study No.	First author (year)	Genes	rs ID (Position)	Ref>Alt allele	Genetic association study					
					Outcome type	Outcome measure	Group comparison	Sample size	Measuring value / Effect size	*p*-value
**1**	**Cao (2020)**	*ABCC2* (*MRP2*)	rs717620 (c.-24)	C>T	PK	$AUC_{0\text{-}72h}$	CT/TT vs. CC	13 vs. 25	402.8 (326.7–438.7) vs. 570.3 (375.8–750.1) μg·h/ml[a]	0.011[b]
						$AUC_{0\text{-}inf}$	CT/TT vs. CC	13 vs. 25	433.9 (337.6–531.2) vs. 685.2 (457.5–907.3) μg·h/ml[a]	0.008[b]
						$t_{1/2}$	CT/TT vs. CC	13 vs. 25	11.1 (8.4–16.6) vs. 17.6 (12.1–25.3) h[a]	0.030[b]
						CL/F	CT/TT vs. CC	13 vs. 25	3.0 (2.3–4.2) vs. 1.8 (1.3–2.7) L/h[a]	0.008[b]
						MRT	CT/TT vs. CC	13 vs. 25	12.6 (11.5–16.2) vs. 14.8 (14.2–18.8) h[a]	0.014[b]
**2**	**Allegra (2017)**	*ABCC2* (*MRP2*)	rs2273697 (c.1249)	G>A	PK	$AUC_{0\text{-}24h}$	GA vs. GG	4 vs. 16	351.871 (191.81–507.33) vs. 160.598 (12.07–383.85) μg·h/ml[a]	0.038[b]
						Vd	GA vs. GG	4 vs. 16	0.38 (0.95–0.39) vs. 1.04 (0.17–10.57) L[a]	0.018[b]
		ABCG2 (*BCRP1*)	rs2231142 (c.421)	G>A	PK	T_{max}	GA/AA	NA	β = 0.601 (95%CI: 0.84-4.05)	0.005[c]
		CYP1A1	rs2606345 (c.-27+606)	C>A	PK	C_{trough}	AA	NA	β = 0.681 (95%CI: 4.90-16.70)	0.001[c]
						$t_{1/2}$	CA/AA vs. CC	16 vs. 4	5.87 (1.45–14.12) vs. 1.41 (1.30–2.15)	0.003[b]
						$t_{1/2}$	AA	NA	β = 0.459 (95%CI: 0.16–7.61)	0.042[c]
					Efficacy	SF	CA/AA vs. CC	16 vs. 4	2763 (2111.08–3190.85) vs. 677 (-720.72–3108.72) ng/ml[a]	0.038[b]
						LIC	CA/AA vs. CC	16 vs. 4	9.101 (8.080–11.067) vs. 6.649 (4.059–8.472) mg Fe/g liver d.w.[a]	0.038[b]
						LIC	CA/AA	NA	β = 0.470 (95%CI: 0.56–6.05)	0.021[c]
					ADR	SCr	AA vs. CC/CA	3 vs. 11	0.650 (0.337–0.926) vs. 0.475 (0.421–0.513) mg/dl[a]	0.036[b]

Study No.	First author (year)	Genes	rs ID (Position)	Ref>Alt allele	Genetic association study					
					Outcome type	Outcome measure	Group comparison	Sample size	Measuring value / Effect size	*p*-value
			rs4646903 (c.*1189)	T>C	PK	T_{max}	TC/CC	NA	β = 0.600 (95%CI: 0.61–2.95)	0.005[c]
					ADR	SCr	TC/CC vs. TT	5 vs. 9	0.550 (0.455–0.718) vs. 0.460 (0.402–0.509) mg/dl[a]	0.019[b]
		CYP1A2	rs762551 (c.-9-154)	A>C	Efficacy	SF	CC	NA	β = -0.439 (95%CI: -4200.06 to -440.25)	0.019[c]
						LIC	AC/CC vs. AA	10 vs. 10	10.302 (8.420–11.915) vs. 6.842 (5.613–9.700) mg Fe/g liver d.w.[a]	0.028[b]
						LIC	AC/CC	NA	β = 0.446 (95%CI: 0.31–4.71)	0.027[c]
					ADR	SCr	AC/CC vs. AA	9 vs. 5	0.460 (0.402–0.509) vs. 0.550 (0.455–0.719) mg/dl[a]	0.019[b]
		CYP2D6	rs1135840 (c.1457)	C>G	PK	C_{max}	CG/GG	NA	β = -0.422 (95%CI: -30.91 to -0.45)	0.044[c]
		UGT1A1	rs887829 (c.-364)	C>T	Efficacy	SF	TT vs. CC/CT	3 vs. 17	900 (57.29-1510.04) vs. 2768 (2098.45-3176.90) ng/ml[a]	0.007[b]
						SF	TT	NA	β = -0.617 (95%CI: -3137.92 to -843.06)	0.002[c]
		UGT1A3	rs1983023 (c.-751)	C>T	ADR	GGT	TT vs. CC/CT	3 vs. 10	9 (8-9.5) vs. 13 (9-30) mU/ml	0.049[b]
			rs3806596 (c.-66)	A>G	Efficacy	SF	GG vs. AA/AG	4 vs. 16	677 (239.60–1162.90) vs. 2810.5 (2287.10–3261.21) ng/ml[a]	< 0.001[b]
4	Mattioli (2015)	*UGT1A1*	rs8175347 (c.-40_-39 dupTA, *UGT1A1*28*)	(TA)6 >(TA)7	PK	C_{ss}	*28/*28 vs. *1/*28 vs. *1/*1	4 vs. 21 vs. 15	NA	0.03[b]
**5**	**Chen (2020)**	*UGT1A1*	rs887829 (c.-364)	C>T	PK	AUC_{0-72h}	CT/TT vs. CC	5 vs. 22	404.96 (348.54–468.65) vs. 532.46 (462.45–743.19) μg·h/ml[a]	< 0.05[b]
						$t_{1/2}$	CT/TT vs. CC	5 vs. 22	13.07 (9.92–14.07) vs. 15.85 (13.22–24.16) h[a]	< 0.05[b]

Study No.	First author (year)	Genes	rs ID (Position)	Ref>Alt allele	Genetic association study					
					Outcome type	Outcome measure	Group comparison	Sample size	Measuring value / Effect size	*p*-value
**6**	**Allegra (2018a)**	*CYP24A1*	rs2248359 (g.3999)	T>C	PK	$AUC_{0\text{-}24h}$	TC/CC vs. TT	7 vs. 2	184.892 (147.18–368.51) vs. 437.339 (370.35–504.33) µg·h/ml[a]	0.040[b]
						$t_{1/2}$	TC/CC vs. TT	7 vs. 2	7.877 (5.70–92.62) vs. 4.780 (4.68–4.88) h[a]	0.040[b]
						Vd	TC/CC vs. TT	7 vs. 2	1319.202 (749.45-10573.86) vs. 386.832 (382.87-390.80) ml[a]	0.040[b]
			rs2585428 (g.8620)	A>G	PK	C_{trough}	AG/GG	NA	β = −0.420 (95%CI: −17.912 to −2.774)	0.012[c]
		CYP27B1	rs10877012 (c.-1261)	G>T	PK	C_{min}	GT vs. GG	2 vs. 7	3.499 (2.38–4.62) vs. 18.322 (8.50–38.45) µg/ml[a]	0.040[b]
			rs4646536 (c.+2838) NM_000785.3 (c.1137-29)	C>T	PK	C_{min}	TT vs. CT	17 vs. 2	18.322 (8.50–38.45) vs. 3.499 (2.38–4.62) µg/ml[a]	0.040[b]
		GC (*VDBP*)	rs7041 (c.1296)	T>G	PK	T_{max}	TG/GG	NA	β = − 0.707 (95%CI: −3.787 to −0.213)	0.033[c]
		VDR	rs10735810 merged into rs2228570 (c.2, Fokl)	T>C	PK	$AUC_{0\text{-}24h}$	CC	NA	β = 0.645 (95%CI: 62.993–258.944)	0.008[c]
						C_{max}	CC	NA	β = 0.544 (95%CI: 5.498–26.571)	0.010[c]
						$t_{1/2}$	CC vs. TT/TC	7 vs. 7	4.879 (4.68–5.70) vs. 8.429 (6.05–92.62) h[a]	0.020[b]
						$t_{1/2}$	TC/CC	NA	β = −0.604 (95%CI: −57.526 to − 20.660)	0.003[c]
						Vd	CC vs. TT/TC	6 vs. 3	390.796 (382.87–749.45) vs. 1611.515 (924.87–10573.86) ml[a]	0.020[b]
						Vd	TC/CC	NA	β = −0.544 (95%CI: − 6704.52 to −1313.24)	0.010[c]
			rs1544410 (c.1024+283, BsmI)	G>A	PK	C_{min}	AA vs. GA	3 vs. 6	4.623 (2.38–13.24) vs. 19.637 (8.50–38.45) µg/ml[a]	0.039[b]

Study No.	First author (year)	Genes	rs ID (Position)	Ref>Alt allele	Genetic association study					
					Outcome type	Outcome measure	Group comparison	Sample size	Measuring value / Effect size	*p*-value
			rs731236 (c.1056, TaqI)	T>C	PK	C_{max}	TC/CC vs. TT	7 vs. 2	17.51 (10.07–26.33) vs. 43.480 (27.45–59.51) μg/ml[a]	0.040[b]
						C_{max}	TC/CC	NA	β = −0.666 (95%CI: −34.207–10.312)	0.004[c]
			rs 7975232 (c.1025-49, ApaI)	C>A	PK	AUC_{0-24h}	AA	NA	β = −0.626 (95%CI: −23.982 to − 0.808)	0.007[c]
						C_{min}	AA vs. CC/CA	5 vs. 4	8.500 (2.38–18.32) vs. 21.151 (13.45–38.45) μg/ml[a]	0.040[b]
						C_{trough}	AA	NA	β = −0.392 (95%CI: −16.832 to −1.375)	0.025[c]
7	Cusato (2015)	*ABCC2* (*MRP2*)	rs2273697 (c.1249)	G>A	PK	C_{trough}	GA vs. GG	20 vs. 34	632.9945 vs. 318.487 ng/ml/kg[a]	0.032[b]
		CYP1A1	rs2606345 (c.-27+606)	C>A	PK	C_{trough}	AA vs. CC/CA	25 vs. 29	317.06 vs 644.67 ng/ml/kg[a]	0.017[b]
						C_{trough} > 20,000 ng/ml	AA	NA	OR = 0.13 (0.02–0.70)	0.017[d]
		CYP1A2	rs2470890 (c.1548)	C>T	PK	C_{trough}	TT vs. CT vs. CC	23 vs. 27 vs. 4	265.6828 vs. 648.6768 vs. 718.2829 ng/ml/kg[a]	0.015[b]
						C_{trough}	TT vs. CC/CT	23 vs. 31	265.6828 vs 648.6768 ng/ml/kg[a]	0.004[b]
						C_{trough} > 20,000 ng/ml	TT	NA	OR = 0.17 (0.03–0.90)	0.037[d]
			rs762551 (c.-9-154)	A>C	PK	C_{trough}	CC vs. AC vs. AA	2 vs. 25 vs. 27	577.8036 vs. 648.6768 vs. 317.06 ng/ml/kg[a]	0.040[b]
						C_{trough}	AC/CC vs. AA	27 vs. 27	648.6768 vs. 317.06 ng/ml/kg[a]	0.014[b]
		UGT1A1	rs887829 (c.-364)	C>T	PK	C_{trough}	TT vs. CC/CT	6 vs. 48	1306.1392 vs. 441.4781 ng/ml/kg[a]	0.045[b]

Study No.	First author (year)	Genes	rs ID (Position)	Ref>Alt allele	Genetic association study					
					Outcome type	Outcome measure	Group comparison	Sample size	Measuring value / Effect size	*p*-value
**8**	**Allegra (2018b)**	*CYP24A1*	rs2248359 (g.3999)	T>C	PK	C_{min}	CC vs. TT/CC	15 vs. 43	0 (0–22.56) vs. 11.160 (0–85.95) µg/ml[a]	0.040[b]
						C_{trough}	CC vs. TT/CC	39 vs. 60	3.556 (0–84.23) vs. 14.050 (0–85.95) µg/ml[a]	0.010[b]
						T_{max}	CC	NA	β = −0.27 (95%CI: −1.16 to −0.05)	0.037[e]
						T_{max}	CC	NA	β = −0.339 (95%CI: −1.61−0.25)	0.008[c]
			rs2585428 (g.8620)	A>G	PK	$AUC_{0\text{-}24h}$	GG vs. AA/AG	17 vs. 47	183.550 (63.76–629.21) vs. 330.551 (76.56–965.54) µg·h/ml[a]	0.021[b]
						$AUC_{0\text{-}24h}$	GG	NA	β = −0.283 (95%CI: −279.09 to −13.81)	0.031[e]
						$AUC_{0\text{-}24h}$	GG	NA	β = −0.283 (95%CI: −279.09 to −13.81)	0.031[c]
						C_{min}	GG vs. AA/AG	17 vs. 47	0 (0–84.23) vs. 0 (0–84.23) µg/ml[a]	0.010[b]
						$t_{1/2}$	GG vs. AA/AG	28 vs. 71	6.479 (1.41–10.47) vs. 8.411 (1.28–47.99) h[a]	0.031[b]
			rs927650 (g.22776)	C>T	PK	C_{min}	TT vs. CC/CT	23 vs. 35	1.148 (0–84.23) vs. 14.430 (0–85.95) µg/ml[a]	0.011[b]
						C_{min}	TT	NA	β = −0.271 (95%CI: −23.98 to −0.81)	0.036[c]
		GC (*VDBP*)	rs7041 (c.1296)	T>G	PK	$AUC_{0\text{-}24h}$ > 360 µg·h/ml	TG/GG	NA	β = 0.178 (95%CI: 0.05–0.66)	0.010[d]
		VDR	rs11568820 (Cdx2)	A>G	PK	$AUC_{0\text{-}24h}$ > 360 µg·h/ml	AG/GG	NA	β = 0.092 (95%CI: 0.01–0.71)	0.022[d]
**9**	**Cusato (2016)**	*ABCG2*	rs13120400 (c.1194+928)	T>C	PK	$AUC_{0\text{-}24h}$ > 360 µg·h/ml	CC	NA	OR = 7.500 (IQR: 1.253–44.885)	0.027[d]
		UGT1A1	rs887829 (c.-364)	C>T	PK	$t_{1/2}$	CT/TT vs. CC	33 vs. 27	8.53 (5.37–11.22) vs. 6.78 (4.99–8.62) h	0.041[b]

Study No.	First author (year)	Genes	rs ID (Position)	Ref>Alt allele	Genetic association study					
					Outcome type	Outcome measure	Group comparison	Sample size	Measuring value / Effect size	*p*-value
		UGT1A3	rs1983023 (c.-751)	C>T	PK	$AUC_{0\text{-}24h}$	CT/TT vs. CC	50 vs. 10	254.47 (170.23–445.26) vs. 639.08 (351.90–804.51) µg·h/ml[a]	0.002[b]
						C_{max}	CT/TT vs. CC	50 vs. 10	21.08 (16.89–34.40) vs. 43.81 (22.94–61.29) µg/ml[a]	0.009[b]
						$t_{1/2}$	TT vs. CC/CT	20 vs. 40	6.19 (4.88–9.31) vs. 8.39 (5.52–11.01) h[a]	0.029[b]
						T_{max}	CT/TT vs. CC	50 vs. 10	4 (4–6) vs. 4 (2–4) h[a]	0.029[b]
						Vd	CT/TT vs. CC	50 vs. 10	952.64 (592.14–1515.85) vs. 550.17 (338.17–904.46) ml[a]	0.021[b]
			rs3806596 (c.-66)	A>G	PK	$AUC_{0\text{-}24h}$	GG vs. AA/AG	13 vs. 47	466.39 (344.55–770.91) vs. 249.87 (168.78–444.97) µg·h/ml[a]	0.001[b]
						$AUC_{0\text{-}24h}$ > 360 µg·h/ml	GG	NA	OR = 10.000 (IQR: 2.287–43.728)	0.002[d]
						$AUC_{0\text{-}24h}$ < 250 µg·h/ml	GG	NA	OR = 0.080 (IQR: 0.010–0.664)	0.019[d]
						C_{max}	GG vs. AA/AG	13 vs. 47	38.86 (24.68–55.85) vs. 19.90 (16.50–34.19) µg/ml[a]	0.004[b]
						$t_{1/2}$	AG/GG vs. AA	45 vs. 15	8.37 (5.37–10.33) vs. 5.81 (4.32–8.62) h[a]	0.043[b]
						T_{max}	GG vs. AA/AG	13 vs. 47	4 (4-6) vs. 4 (2–4) h[a]	0.042[b]
						Vd	GG vs. AA/AG	13 vs. 47	588.72 (353.27–758.72) vs. 966.31 (636.73–1541.83) ml[a]	0.005[b]
**10**	**Allegra (2019)**	*CYP27B1*	rs10877012 (c.-1261)	G>T	Efficacy	Liver stiffness	GT/TT vs. GG	47 vs. 58	6.30 (4.90–9.00) vs. 5.15 (4.30–6.60) mean kPa/year[a]	0.010[b]
						Liver stiffness	GT/TT	NA	β = 0.193 (95%CI: 0.072-1.806)	0.034[c]

Study No.	First author (year)	Genes	rs ID (Position)	Ref>Alt allele	Genetic association study					p-value
					Outcome type	Outcome measure	Group comparison	Sample size	Measuring value / Effect size	*p*-value
						Liver stiffness < 7 kPa	GT/TT	NA	Exp(*B*) = 0.297 (95%CI: 0.11-0.80)	0.016[d]
		CYP2D6	rs1135840 (c.1457)	G>C	Efficacy	Liver T2*	GG	NA	β = 0.163 (95%CI: 0.362-6.041)	0.028[c]
		UGT1A1	rs887829 (c.-364)	C>T	Efficacy	Liver stiffness	CT/TT vs. CC	62 vs. 43	6.15 (4.90–8.93) vs. 5.10 (4.20–6.40) mean kPa/year[a]	0.005[b]
						Liver stiffness	CT/TT	NA	β = 0.357 (95%CI: 0.818-2.689)	< 0.001[c]
						Liver T2* > 6.3 ms	CT/TT	NA	Exp(*B*) = 5.080 (95%CI: 1.33-19.48)	0.018[d]
		UGT1A3	rs1983023 (c.-751)	C>T	Efficacy	Liver stiffness	TT vs. CC/CT	32 vs. 73	4.95 (4.13–8.94) vs. 6.10 (4.85–8.53) mean kPa/year[a]	0.009[b]
		GC (*VDBP*)	rs7041 (c.1296)	T>G	Efficacy	Liver stiffness	TG/GG vs. TT	61 vs. 44	5.75 (4.90–8.53) vs. 5.23 (4.21–6.80) mean kPa/year[a]	0.042[b]
		VDR	rs1544410 (c.1024+283, BsmI)	G>A	Efficacy	Liver stiffness	AA vs. AA/GA	22 vs. 83	4.70 (3.69–6.45) vs. 5.70 (4.80–8.75) mean kPa/year[a]	0.015[b]
			rs731236 (c.1056, TaqI)	T>C	Efficacy	Liver stiffness	CC vs. TT/TC	20 vs. 85	4.70 (3.56–6.70) vs. 5.75 (4.79–8.53) mean kPa/year[a]	0.016[b]
11	Allegra (2018c)	*ABCG2*	rs13120400 (c.1194+928)	T>C	Efficacy	Cardiac T2*	TC/CC	NA	β = −0.180 (95%CI: −8.996 to −0.192)	0.041[c]
			rs2231142 (c.421)	G>A	Efficacy	Cardiac T2*	GA vs. GG	19 vs. 86	30.20 (15.70–43.30) vs. 42.50 (35.90–47.55) ms mean/year[a]	0.008[b]
						Cardiac T2*	GA	NA	β = −0.274 (95%CI: −14.736 to −3.436)	0.002[c]
		CYP1A1	rs4646903 (c.*1189)	T>C	Efficacy	Cardiac T2*	CC vs. TT/TC	6 vs. 99	27.7 (15.91–39.88) vs. 42.3 (32.40–47.46) ms mean/year[a]	0.038[b]

Study No.	First author (year)	Genes	rs ID (Position)	Ref>Alt allele	Genetic association study					
					Outcome type	Outcome measure	Group comparison	Sample size	Measuring value / Effect size	*p*-value
		CYP24A1	rs2585428 (g.8620)	A>G	Efficacy	Cardiac T2*	GG vs. AA/AG	29 vs. 76	36.10 (22.75–42.90) vs. 43.60 (35.95–47.70) ms mean/year[a]	0.021[b]
			rs927650 (g.22776)	C>T	Efficacy	Cardiac T2*	TT	NA	β = 0.179 (95%CI: 0.217–10.355)	0.041[c]
		VDR	rs731236 (c.1056, TaqI)	T>C	Efficacy	Cardiac T2*	CC vs. TT/TC	20 vs. 85	45.80 (39.28−49.40) vs. 39.80 (24.99−46.10) ms mean/year[a]	0.006[b]
					Efficacy	Cardiac T2*	TC/CC	NA	β = 0.191 (95%CI: 0.521–9.674)	0.029[c]
12	Lee (2013)	*ABCC2* (*MRP2*)	rs717620 and/or rs369192412 (-1774delG)	c.-24C>T and/or -1774 G>del	ADR	Hepato toxicity	Haplotypes containing c.-24C>T and/or -1774 G>del vs. Haplotypes containing wild–type	34 vs. 64	OR = 7.17 (95%CI: 1.79–28.67)	0.005[d]
		UGT1A1	rs4148323 (c.211, *UGT1A1*6*)	G>A	ADR	Creatinine elevation	AA vs. GG/GA	6 vs. 92	OR = 14.17 (95%CI: 1.34–150.35)	0.028[d]

Bold indicated the studies included in meta-analysis. NA, not available; SF, serum ferritin; Ref>Alt allele, Reference allele and alternative allele.

[a] Median (IQR); [b] Analyzed by using Mann-Whitney *U* test or Kruskal-Wallis test; [c] Analyzed by using multivariate linear regression analysis; [d] Analyzed by using multivariate logistic regression analysis; [e] Analyzed by using univariate linear regression.

AUC, area under the plasma concentration-time curve; $AUC_{0\text{-}24h}$, area under the plasma concentration-time curve from 0 to 24 h; $AUC_{0\text{-}72h}$, area under the plasma concentration-time curve from 0 to 72 h; CL/F, apparent oral clearance; C_{max}, maximum plasma concentration; C_{min}, minimum plasma concentration; C_{trough}, Trough concentration; LIC, Liver iron concentration; LS, Liver stiffness; MRT, Mean residence time; SCr, Serum creatinine; SF, serum ferritin; $t_{1/2}$, half-life; T_{max}, time to reach maximum concentration; Vd, volume of distribution.

Supplementary Table S7 Meta-analysis results of the associations between genetic polymorphisms and deferasirox outcomes.

Genes/ LD groups	SNPs	Outcome	Included Study No.	Group comparison	Association result		Heterogeneity		
					ROM [95% CI]	*p*-value	I^2	*Q*-test *p*-value	Model
ABCC2 (*MRP2*)	rs2273697 (G>A)	AUC	1, 2, 5, 9	GA/AA vs. GG	1.10 [0.80, 1.52]	0.54	37%	0.19	Random
		Chinese	1, 5	GA/AA vs. GG	0.91 [0.69, 1.21]	0.52	4%	0.31	Random
		Caucasian	2, 9	GA/AA vs. GG	1.48 [0.97, 2.27]	0.07	0%	0.68	Random
		C_{max}	1, 2, 5, 9	GA/AA vs. GG	1.23 [1.06, 1.43]	**0.007**	11%	0.34	Random
		Chinese	1, 5	GA/AA vs. GG	1.17 [1.01, 1.35]	**0.04**	0%	0.39	Random
		Caucasian	2, 9	GA/AA vs. GG	1.52 [1.11, 2.08]	**0.008**	0%	0.55	Random
		T_{max}	1, 2, 9	GA/AA vs. GG	1.03 [0.84, 1.27]	0.77	0%	0.79	Random
		Chinese	1	GA/AA vs. GG	0.96 [0.72, 1.29]	0.79	NA	NA	NA
		Caucasian	2, 9	GA/AA vs. GG	1.11 [0.83, 1.48]	0.50	0%	0.87	Random
		$t_{1/2}$	1, 2, 5, 9	GA/AA vs. GG	0.90 [0.72, 1.13]	0.36	40%	0.17	Random
		Chinese	1, 5	GA/AA vs. GG	1.10 [0.80, 1.51]	0.57	0%	0.50	Random
		Caucasian	2, 9	GA/AA vs. GG	0.80 [0.59, 1.08]	0.15	57%	0.13	Random
		Vd	2, 9	GA/AA vs. GG	0.48 [0.36, 0.63]	**< 0.00001**	61%	0.11	Fixed
ABCG2 (*BCRP1*)	rs13120400 (T>C)	Vd	2, 9	CC vs. TT/TC	1.42 [0.92, 2.19]	0.12	0%	0.39	Fixed
UGT1A1	rs887829 (C>T)	AUC	2, 5, 9	CT/TT vs. CC	0.99 [0.57, 1.71]	0.97	81%	0.005	Random
		Chinese	5	CT/TT vs. CC	0.70 [0.52, 0.95]	**0.02**	NA	NA	NA
		Caucasian	2, 9	CT/TT vs. CC	1.21 [0.65, 2.27]	0.55	69%	0.07	Random
		C_{max}	2, 5, 9	CT/TT vs. CC	1.02 [0.71, 1.46]	0.92	71%	0.03	Random
		Chinese	5	CT/TT vs. CC	0.82 [0.63, 1.06]	0.13	NA	NA	NA
		Caucasian	2, 9	CT/TT vs. CC	1.18 [0.80, 1.74]	0.41	51%	0.15	Random
		$t_{1/2}$	2, 5, 9	CT/TT vs. CC	1.00 [0.67, 1.48]	0.99	71%	0.03	Random
		Chinese	5	CT/TT vs. CC	0.70 [0.49, 1.00]	0.05	NA	NA	NA
		Caucasian	2, 9	CT/TT vs. CC	1.22 [0.98, 1.53]	0.08	0%	0.86	Random
UGT1A3	rs1983023 (C>T)	$t_{1/2}$	2, 9	TT vs. CC/CT	0.84 [0.65, 1.07]	0.16	0%	0.72	Fixed
	rs3806596 (A>G)	AUC	2, 5	AG/GG vs. AA	0.78 [0.60, 0.99]	**0.04**	0%	0.73	Random
		C_{max}	2, 5	AG/GG vs. AA	0.89 [0.70, 1.14]	0.37	0%	0.87	Random
		$t_{1/2}$	2, 5, 9	AG/GG vs. AA	1.18 [0.94, 1.48]	0.16	0%	0.69	Random
		Chinese	5	AG/GG vs. AA	1.02 [0.67, 1.55]	0.92	NA	NA	NA
		Caucasian	2, 9	AG/GG vs. AA	1.25 [0.95, 1.65]	0.11	0%	0.77	Random
		SF	2, 10	GG vs. AA/AG	0.39 [0.23, 0.67]	**0.0006**	0%	0.5	Fixed
CYP1A1	rs2606345 (C>A)	$t_{1/2}$	2, 5	CA/AA vs. CC	2.01 (0.46, 8.75)	0.35	86%	0.007	Random
CYP24A1	rs2248359 (T>C)	AUC	6, 8	TC/CC vs. TT	0.60 [0.35, 1.02]	0.06	0%	0.77	Fixed
		C_{min}	6, 8	CC vs. TT/TC	0.26 [0.08, 0.93]	**0.04**	0%	0.56	Fixed
		C_{trough}	6, 10	CC vs. TT/TC	0.50 [0.29, 0.87]	**0.01**	0%	0.42	Fixed
		$t_{1/2}$	6, 8	TC/CC vs. TT	0.78 [0.49, 1.24]	0.30	87%	0.006	Fixed
	rs2585428 (A>G)	AUC	6, 8	GG vs. AA/AG	0.66 [0.31, 1.44]	0.30	0%	0.85	Fixed
		C_{min}	6, 8	GG vs. AA/AG	0.84 [0.24, 2.90]	0.78	0%	0.93	Fixed
		C_{trough}	6, 10	GG vs. AA/AG	0.47 [0.35, 0.63]	**< 0.00001**	0%	0.61	Fixed
		$t_{1/2}$	6, 8	GG vs. AA/AG	0.44 [0.23, 0.83]	**0.01**	41%	0.19	Fixed
	rs927650 (C>T)	C_{min}	6, 8	TT vs. CC/CT	0.77 [0.30, 1.96]	0.58	0%	0.77	Fixed

Genes/ LD groups	SNPs	Outcome	Included Study No.	Group comparison	Association result		Heterogeneity		
					ROM [95% CI]	*p*-value	I^2	*Q*-test *p*-value	Model
CYP27B1	rs10877012 (G>T)	C_{min}	6, 8	GT/TT vs. GG	0.78 [0.42, 1.43]	0.42	69%	0.07	Fixed
	rs4646536 (C>T)	C_{min}	6, 8	TT vs. CC/CT	1.49 [0.83, 2.69]	0.18	69%	0.07	Fixed
VDR	rs1544410 (Bsml G>A)	C_{min}	6, 8	AA vs. GG/GA	0.73 [0.40, 1.32]	0.30	0%	0.48	Fixed
	rs731236 (TaqI T >C)	C_{max}	6, 8	TC/CC vs. TT	0.77 [0.54, 1.09]	0.15	0%	0.52	Fixed
	rs7975232 (ApaI C>A)	C_{min}	6, 8	AA vs. CC/CA	0.74 [0.40, 1.35]	0.32	0%	0.48	Fixed
LD_c	rs10929302 or rs887829	AUC	1, 2, 5, 9	Alt carrier vs Homo Ref[a]	0.99 [0.66, 1.48]	0.94	72%	0.01	Random
		Chinese	1, 5	Alt carrier vs Homo Ref[a]	0.78 [0.58, 1.05]	0.11	18%	0.27	Random
		Caucasian	2, 9	Alt carrier vs Homo Ref[a]	1.21 [0.65, 2.27]	0.55	69%	0.07	Random
		C_{max}	1, 2, 5, 9	Alt carrier vs Homo Ref[a]	1.01 [0.78, 1.31]	0.93	56%	0.08	Random
		Chinese	1, 5	Alt carrier vs Homo Ref[a]	0.86 [0.69, 1.08]	0.19	0%	0.43	Random
		Caucasian	2, 9	Alt carrier vs Homo Ref[a]	1.18 [0.80, 1.74]	0.41	51%	0.15	Random
LD_d	rs3755319 or rs3806596	AUC	1[b], 2, 5	Alt carrier vs Homo Ref[c]	0.83 [0.68, 1.01]	0.07	0%	0.61	Random
		Chinese	1[b], 5	Alt carrier vs Homo Ref[c]	0.83 [0.67, 1.02]	0.08	0%	0.32	Random
		Caucasian	2	Alt carrier vs Homo Ref[c]	0.85 [0.47, 1.53]	0.59	NA	NA	NA
		C_{max}	1[b], 2, 5	Alt carrier vs Homo Ref[c]	0.95 [0.81, 1.13]	0.58	0%	0.77	Random
		Chinese	1[b], 5	Alt carrier vs Homo Ref[c]	0.96 [0.81, 1.14]	0.63	0%	0.47	Random
		Caucasian	2	Alt carrier vs Homo Ref[c]	0.93 [0.56, 1.52]	0.76	NA	NA	NA

Alt carrier, alternative allele carrier; CI, confidence interval; Homo Ref, homozygous reference allele; LD, Linkage disequilibrium; LD_c, LD group C; LD_d, LD group D; LD_e, LD group E; NA, not applicable; ROM, Ratio of means; SNPs, Single nucleotide polymorphisms.

Bold indicated a statistically significant difference at *p*-value < 0.05.

[a] GA/AA genotypes vs. GG genotype of rs10929302 or CT/TT genotypes vs. CC genotype of rs887829.

[b] Only AA and AC genotypes of *UGT1A3* rs3755319 in this study were used for the meta-analysis due to unavailable data of interquartile range (IQR) of CC genotype.

[c] AC genotype vs. AA genotype of rs3755319 or AG/GG genotypes vs. AA genotype of rs3806596.
